# An integrated metabo-lipidomics profile of induced sputum for the identification of novel biomarkers in the differential diagnosis of asthma and COPD

**DOI:** 10.1186/s12967-024-05100-2

**Published:** 2024-03-23

**Authors:** Serena Correnti, Mariaimmacolata Preianò, Fabia Gamboni, Daniel Stephenson, Corrado Pelaia, Girolamo Pelaia, Rocco Savino, Angelo D’Alessandro, Rosa Terracciano

**Affiliations:** 1 Department of Health Sciences, Magna Græcia University, 88100 Catanzaro, Italy; 2 Department of Biochemistry and Molecular Genetics, University of Colorado Anschutz Medical Campus, Aurora, CO 80045 USA; 3 Department of Medical and Surgical Sciences, Magna Græcia University, 88100 Catanzaro, Italy; 4 Department of Experimental and Clinical Medicine, Magna Græcia University, 88100 Catanzaro, Italy

**Keywords:** Induced sputum, Asthma, COPD, Metabolomics, Lipidomics, Biomarkers, Mass spectrometry

## Abstract

**Background:**

Due to their complexity and to the presence of common clinical features, differentiation between asthma and chronic obstructive pulmonary disease (COPD) can be a challenging task, complicated in such cases also by asthma–COPD overlap syndrome. The distinct immune/inflammatory and structural substrates of COPD and asthma are responsible for significant differences in the responses to standard pharmacologic treatments. Therefore, an accurate diagnosis is of central relevance to assure the appropriate therapeutic intervention in order to achieve safe and effective patient care. Induced sputum (IS) accurately mirrors inflammation in the airways, providing a more direct picture of lung cell metabolism in comparison to those specimen that reflect analytes in the systemic circulation.

**Methods:**

An integrated untargeted metabolomics and lipidomics analysis was performed in IS of asthmatic (n = 15) and COPD (n = 22) patients based on Ultra-High-Pressure Liquid Chromatography-Mass Spectrometry (UHPLC-MS) and UHPLC–tandem MS (UHPLC-MS/MS). Partial Least Squares-Discriminant Analysis (PLS-DA) was applied to resulting dataset. The analysis of main enriched metabolic pathways and the association of the preliminary metabolites/lipids pattern identified to clinical parameters of asthma/COPD differentiation were explored. Multivariate ROC analysis was performed in order to determine the discriminatory power and the reliability of the putative biomarkers for diagnosis between COPD and asthma.

**Results:**

PLS-DA indicated a clear separation between COPD and asthmatic patients. Among the 15 selected candidate biomarkers based on Variable Importance in Projection scores, putrescine showed the highest score. A differential IS bio-signature of 22 metabolites and lipids was found, which showed statistically significant variations between asthma and COPD. Of these 22 compounds, 18 were decreased and 4 increased in COPD compared to asthmatic patients. The IS levels of Phosphatidylethanolamine (PE) (34:1), Phosphatidylglycerol (PG) (18:1;18:2) and spermine were significantly higher in asthmatic subjects compared to COPD.

**Conclusions:**

This is the first pilot study to analyse the IS metabolomics/lipidomics signatures relevant in discriminating asthma *vs* COPD. The role of polyamines, of 6-Hydroxykynurenic acid and of d-rhamnose as well as of other important players related to the alteration of glycerophospholipid, aminoacid/biotin and energy metabolism provided the construction of a diagnostic model that, if validated on a larger prospective cohort, might be used to rapidly and accurately discriminate asthma from COPD.

**Graphical Abstract:**

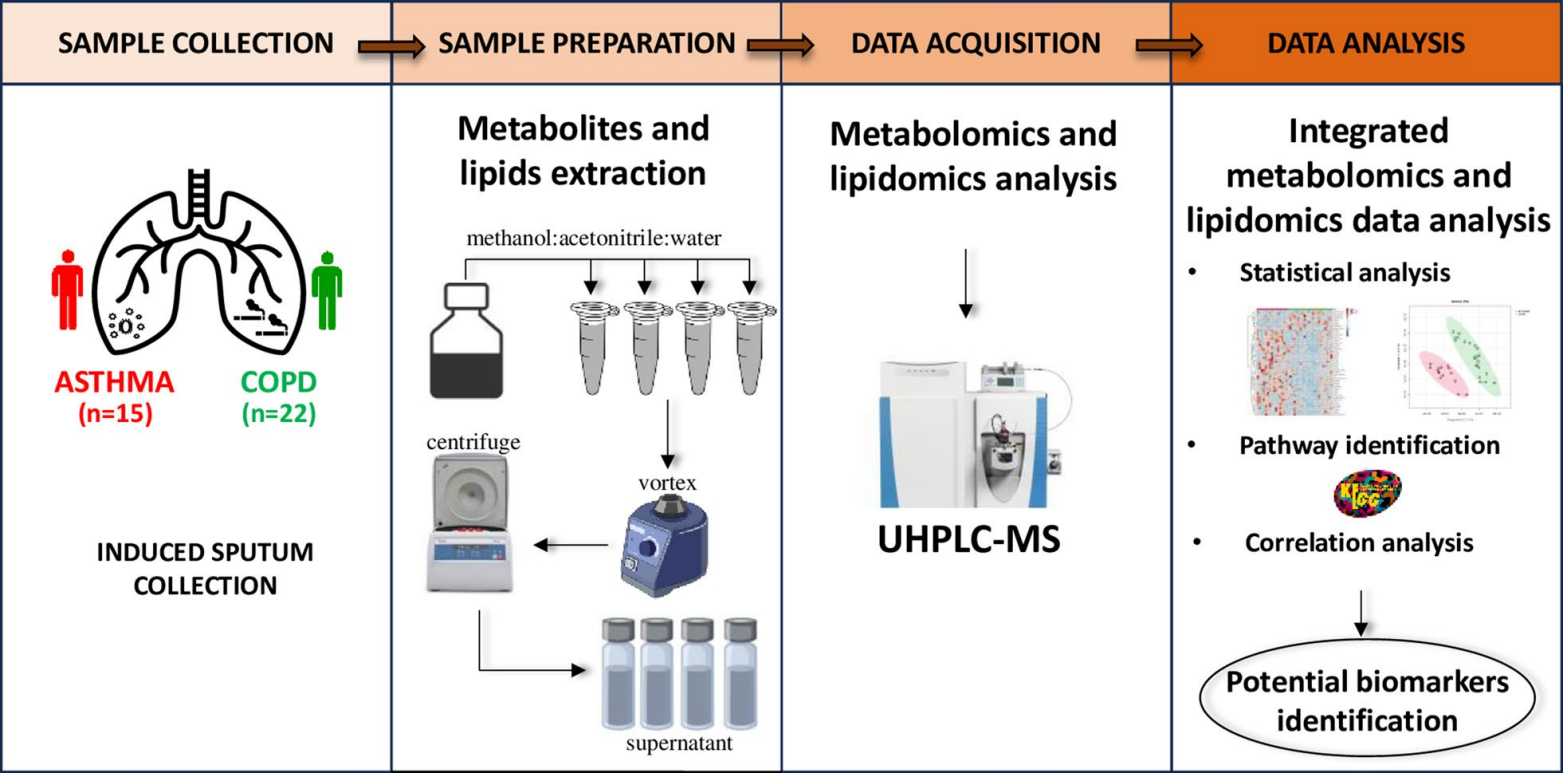

**Supplementary Information:**

The online version contains supplementary material available at 10.1186/s12967-024-05100-2.

## Background

Asthma and chronic obstructive pulmonary disease (COPD) are the most common lung diseases, with COPD the third leading cause of death worldwide [1]. The global prevalence is approximately 3.6% for asthma and 3.9% for COPD [2]. Currently, differentiation of asthma and COPD is mainly based on clinical and lung function parameters. Although asthma and COPD are two etiological different diseases with different pathophysiology and different patient characteristics, patients can sometimes present similar clinical features of both diseases and invasive diagnostic methods are required to improve diagnostic accuracy [3]. In older subjects, the predicted forced expiratory volume in 1 s (FEV1) falls to 50% in both asthma and COPD patients over 60 years old, therefore it could be difficult to discriminate between COPD and asthma [4]. Sometimes, patients with asthma might develop COPD later in life as well as patients with COPD can show clinical characteristics usually observed in asthma [5]. To further complicate this picture, asthma and COPD can occur together for such patients, and, in clinical practice, the term asthma-COPD overlap (ACO) is used to describe these cases [6]. Therefore, differentiating between these multifactorial pulmonary diseases can be difficult, posing many problems in the clinical setting and management, especially in case of misdiagnosis that may lead to inadequate treatment. Finally, yet importantly, the problem of underdiagnoses in COPD and asthma lead patients to begin therapy very late with increased risk of acute exacerbations and early death [7, 8].

Metabolomics, as well as other MS-based ‘omics, with the combined use of high-throughput technologies and multivariate statistical approaches, represents a valuable tool in the study of biochemical and metabolic processes underlying lung respiratory diseases [9–12]. In fact, metabolism dysregulation plays an important role in the development of both acute and chronic lung diseases. Additionally, alterations in metabolism may influence not only the severity and the progression of diseases but also patient response to a specific therapy [13]. Asthma and COPD metabolomics made great strides in the past two decades, highlighting main metabolic arrangements and providing an accurate characterization of metabolic pathways associated to asthma and COPD [9, 10]. However, the use of metabolomics as diagnostic tool to be translated into clinical practice, still remains a research gap to be filled.

Recent progress in MS-based integrated metabolomics and lipidomics has opened new horizons in the field of biomarkers discovery and translational medicine by establishing a more inclusive pattern profiling of small molecules, which can better characterize metabolic functions [14, 15]. In particular, biomarkers discovery from integrated metabolomics and lipidomics data will not only help the stratification of different patient cohorts but will also provide a potent tool for comparative analysis with high diagnostic value that could improve patient management [16–18].

Up to date, only three untargeted LC-MS studies have explored the metabolomics differences between asthma and COPD based on serum [19] and urine [20, 21], which are two important and non-invasive samples reflecting analytes in the systemic circulation. It would be beneficial, however, to analyze specific biomarkers also at a cellular level in the more proximal site of inflammation. Induced sputum (IS) contains specific information related to inflammatory cells and specific mediators of these two respiratory disorders and closely reflects the pathophysiology of these two diseases; therefore, it is widely recognized as an ideal source of biomarkers [11, 12, 22].

Comparison of the wide arrays of metabolites and lipids abundances between asthma and COPD might therefore unmask specific inflammatory and immune response associated to asthma and COPD, enabling the establishment of diagnostic models to be translated into the differential clinical management of these two diseases.

In the present study, an UHPLC-MS and UHPLC-MS/MS based metabolomics and lipidomics untargeted approach was used to analyze for first time the metabolomics profiles of IS from asthmatic and COPD patients. In particular, we performed a multi-tiered analysis of the metabolomics and lipidomics data obtained from IS comparing these specific biosignatures between the two groups. This integrated approach, by comparing a wide array of metabolites and lipids of a such rich informative fluid of the respiratory airways, not only opens new horizons for the discovery of novel putative biomarkers which can better differentiate asthma from COPD, but also implements the patients’ choice for appropriate and specific therapies.

## Materials and methods

### Study population

A total of 15 asthmatic patients and 22 COPD patients were recruited in accordance with the principles of the Declaration of Helsinki, and the study was approved by the Institutional Ethical Committee Board of MAGNA GRAECIA UNIVERSITY and MATER DOMINI HOSPITAL (Approval number 219, date of approval:16 November 2016). All patients were enrolled after giving their informed written consent. For all participants an informed consent was provided before enrollment. Asthma and COPD were diagnosed according to the Global Initiative for Asthma guidelines [23] and the Global Initiative for Chronic Obstructive Lung Disease guidelines [24], respectively. Specifically, clinically diagnosed asthmatic subjects showed evidence of airway hyperresponsiveness with 20% drop in FEV1 after a provocative dose of methacholine < 2.5 mg. Subjects treated with oral corticosteroids and those affected by respiratory infection in one month before enrollement were excluded. COPD and asthmatic patients with important comorbidities including heart, liver, kidney pathology, or any other respiratory disease that could possibly influence this study were also excluded. The clinical characteristics of the subjects recruited in this study, including demographic characteristics, smoking status and spirometry function were collected and are summarized in Table 1.

**Table 1:** Clinical features of the asthmatic and COPD patients enrolled in the study

Patients’ characteristics		Asthma	COPD
Demographic data	Number of participants	15	22
	Age (years)	53,7 ± 14,3	68,7** ± 7,1
	Sex (male/female)	5/10	20/2
Smoking status	Never-smokers	10	1
	Ex-smokers	4	15
	Current-smokers	1	6
Lung function	FEV1 (% predicted)^a^	75,9 ± 16,4	43,8*** ± 21,2
	FVC (% predicted)^b^	87,5 ± 17,3	72,3* ± 12,5
	FEV1/FVC	0,88 ± 0,1	0,62* ± 0,2

Data are expressed as numbers or mean ± standard deviation

^a^Forced expiratory volume in 1 s percentage predicted

^b^Forced vital capacity percentage predicted

^*^p-value < 0.01 (asthma *vs* COPD)

^**^p-value < 0.001 (asthma *vs* COPD)

^***^p-value < 0.0001

### Induced sputum collection and processing

IS samples were collected and processed in accordance with a previous study by Terracciano et al*.* [11]. Sputum induction was carried out according to the guidelines of the European Respiratory Society Task Force, with a modification of the standard procedure of induction. Specifically, besides the administration of 4.5% hypertonic saline at 5 min intervals for a maximum of 20 min, participants were instructed to rinse their mouths with water and blow their noses after each inhalation to prevent contamination from saliva and postnasal drip. Only the mucoid components of sputum were selected in order to minimize the influence of salivary contamination. Samples were treated with PBS to which DTT had been added to a final concentration of 5 mM DTT. Then, a protease inhibitor cocktail (P8340 Sigma-Aldrich) was added to the sputum sample (22.5 mL/g of sputum). The tubes, containing the samples, were then placed on a bench roller for 30 min at room temperature and the contents were filtered through a 70 mm mesh and subsequently centrifuged at 400 g for 10 min at 4 °C, to separate the cells from the fluid phase. The supernatants were separated from the pellets and re-centrifuged at 12,000 g, for 10 min at 4 °C, aliquoted and stored at – 80 °C until use. Protein concentration in sputum samples was determined by BCA assay (Pierce^™^ BCA Protein Assay Kit, 23225, Thermo Fisher) and data normalization to protein concentration was performed.

### Sample preparation for metabolites and lipids extraction

Metabolomics and lipidomics analysis were carried out at the University of Colorado Anschutz Medical Campus Metabolomics Core. First, IS samples were thawed and were treated with a solution of chilled 5:3:2 methanol:acetonitrile:water (v/v/v) at 1:25 ratio, vortexed for 30 min at 4 °C and centrifugated for 10 min at 18,000 rcf at 4 °C, in order to extract metabolites and lipids. After centrifugation, the supernatants were carefully collected and used for metabolomics and lipidomics analysis according to previous studies [18, 25, 26].

### UHPLC-MS metabolomics

Metabolomics analyses of IS samples were conducted using a Vanquish UHPLC combined with a high-resolution Q Exactive mass spectrometer (Thermo Fisher, Bremen, Germany) in negative and positive polarity modes in accordance to previous studies [18, 25]. In the case of negative polarity mode, 5 μL of extracts were injected into the UHPLC system and resolved on a Kinetex C18 column (150 × 2.1 mm, 1.7 μm, Phenomenex, Torrance, CA, USA) at 450 μL/min through a 5 min gradient from 0 to 100% organic solvent B (mobile phases: A = 95% water, 5% acetonitrile, 1 mM ammonium acetate; B = 95% acetonitrile, 5% water, 1 mM ammonium acetate). Solvent gradient: 0–0.5 min 0% B, 0.5–1.1 min 0–100% B, 1.1–2.75 min hold at 100% B, 2.75–3 min 100–0% B, 3–5 min hold at 0% B. In the case of positive polarity mode, 5 μL of extracts were injected into the UHPLC system and resolved on a Kinetex C18 column (150 × 2.1 mm, 1.7 μm, Phenomenex, Torrance, CA, USA) at 450 μL/min through a 5 min gradient from 5 to 95% organic solvent B (mobile phases: A = water, 0.1% formic acid; B = acetonitrile, 0.1% formic acid). Solvent gradient: 0–0.5 min 5% B, 0.5–1.1 min 5–95% B, 1.1–2.75 min hold at 95% B, 2.75–3 min 95–5% B, 3–5 min hold at 5% B. The UHPLC system was coupled online with a Q Exactive mass spectrometer, which operated in Full MS mode with a resolution of 70,000 in the 60–900 *m/z* range. The instrument was set to a 4 kV spray voltage, 15 sheath gas, and 5 auxiliary gas, with negative or positive ion mode (separated runs). Calibration was conducted prior to analysis using the Pierce^™^ Positive and Negative Ion Calibration Solutions (Thermo Fisher Scientific). Samples were analyzed in randomized order and technical mixtures as quality controls were used to qualify instrument performance. Quality controls (generated by mixing 10 μL of all samples tested in this study) were injected every 10 runs to warrant technical reproducibility, as demonstrated by determining coefficients of variation (CV, calculated by dividing standard deviations for each metabolite by the mean across all tech mixes) for each reported metabolite for chromatographic retention times and peak areas. A coefficient of variation < 20% was used as threshold. Metabolomics data were collected using Thermo Fisher Scientific Xcalibur software (v.4.1.31.9). LC-MS raw files were converted to mzXML file format using MSconvert v.3.0.20315-7da487568 (ProteoWizard). Metabolite assignments were performed using accurate intact mass (sub-10 ppm), isotopologue distributions, and comparing retention time/spectra to an in-house standard compound library (MSMLS, IROA Technologies, NJ, USA) using MAVEN (Princeton, NJ, USA) (without performing MS/MS methods). The MAVEN software platform provides tools for peak picking (based on peak intensity, quality control consistency, retention time and ^13^C abundance), feature detection and metabolite assignment using the Kyoto Encyclopedia of Genes and Genomes (KEGG) pathway database.

### UHPLC-MS lipidomics

Lipidomics analyses of IS were performed using a Vanquish UHPLC system coupled online to a high-resolution Q Exactive mass spectrometer (Thermo Fisher, Bremen, Germany) in negative and positive polarity modes in accordance to previous studies [18, 26]. Samples were separated over an ACQUITY HSS T3 column (2.1 × 150 mm, 1.8 µm particle size (Waters, MA, USA). In the case of negative polarity mode, an aqueous phase (A) of 25% acetonitrile and 5 mM ammonium acetate and a mobile phase (B) of 50% isopropanol, 45% acetonitrile, and 5 mM ammonium acetate. Solvent gradient: 0–1 min 25% B and 0.3 mL/min, 1–2 min 25–50% B and 0.3 mL/min, 2–8 min 50–90% B and 0.3 mL/min, 8–10 min 90–99% B and 0.3 mL/min, 10–14 min hold at 99% B and 0.3 mL/min, 14–14.1 min 99–25% B and 0.3 mL/min, 14.1–16.9 min hold at 25% B and 0.4 mL/min, and 16.9 17 min hold at 25% B and resume flow of 0.3 mL/min. In the case of positive polarity mode, an aqueous phase (A) of 25% acetonitrile and 5 mM ammonium acetate and a mobile phase (B) of 90% isopropanol, 10% acetonitrile, and 5 mM ammonium acetate. Solvent gradient: 0–10 min 30–100% B and 0.325 mL/min; 10–12 min 100% B and 0.325 mL/min; 12–12.5 min 100–30% B and 0.4 mL/min; 12.5–14.9 min 30% B and 0.4 mL/min; 14.9 15 min 30% B and 0.325 mL/min. The Q Exactive mass spectrometer (Thermo Fisher Scientific, San Jose, CA, USA) was operated independently in positive or negative ion mode, scanning in Full MS mode (2 μscans) from 150 to 1500 m/z with a resolution of 70,000, 4 kV spray voltage, 45 sheath gas, and 15 auxiliary gas. For discovery mode untargeted lipidomics, dd-MS2 was performed at 17,500 resolution, AGC target = 1 × 10^5^, maximum IT = 50 ms, and stepped NCE of 25, 35 for positive mode, and 20, 24, and 28 for negative mode. Calibration was conducted prior to analysis using the Pierce^™^ Positive and Negative Ion Calibration Solutions (Thermo Fisher Scientific). Samples were analyzed in randomized order and technical mixture or quality controls (generated by mixing 10 μL of all samples tested in this study) were injected every 10 runs and were used to qualify instrument performance (CV < 20%).

Discovery mode analysis was performed with standard workflows using Compound Discoverer 2.1 SP1 (Thermo Fisher Scientific, San Jose, CA, USA). From these analyses, lipid IDs or unique chemical formulae were determined from high-resolution accurate intact mass, isotopic patterns, identification of eventual adducts (e.g., Na^+^ or K^+^, etc.) and MS2 fragmentation spectra against the KEGG pathway, HMDB, ChEBI, and ChEMBL databases. Additional untargeted lipidomics analyses were performed with the software LipidSearch 4.0 (Thermo Fisher, Bremen, Germany), which provides lipid identification on the basis of accurate intact mass, isotopic pattern, and fragmentation pattern to determine lipid class and acyl chain composition [26].

### Statistical analysis and data integration

After the independent collection of metabolomics and lipidomics data from IS samples, distinct files for metabolomics and lipidomics were generated via MAVEN and Compound Discover, respectively, including a list of all the metabolites and lipids identified in the samples. Subsequently, these files were merged together in a single file and used to perform the subsequent integrated data analysis. Statistical analyses were conducted using a two-sided Student’s t-test on peak intensity and the results were considered statistically significant with *p*-values < 0.05. Multivariate analyses were performed using MetaboAnalyst (5.0) and encompassed Partial Least Square-Discriminant Analysis (PLS-DA), Orthogonal partial least squares discriminant analysis (OPLS-DA) and hierarchical clustering analyses. PLS-DA and OPLS-DA were multivariate supervised methods used to minimize data dimensionality and identify variables that exhibit the capacity to distinguish between different groups. Hierarchical Clustering Analyses was performed to visualize the top 50 metabolites and lipids through a heatmap, allowing for a clear representation of their patterns. Additionally, box plot analysis was performed by OriginLab^®^ software (version 7.0, OriginLab Corporation, Northampton, MA, USA) and correlation analysis (Spearman) was carried out in JASP open-source statistical software (Version 0.18.1, https://jasp-stats.org). The correlation analysis aimed to provide valuable insights into the clinical relevance and the potential diagnostic significance of the identified metabolites and lipids. Lastly, ROC (Receiver Operating Characteristic) curve analysis was performed using MetaboAnalyst (5.0), to assess the diagnostic performance of biomarkers and evaluate their ability to discriminate between different groups or conditions.

### Spearman’s correlation analysis

The relationship between significantly altered metabolites (asthma *vs.* COPD) and lung function parameters was explored using Spearman’s correlation analysis, conducted using JASP open-source statistical software (Version 0.18.1, https://jasp-stats.org). In order to investigate the extent to which the altered metabolites were linearly related to FEV1 and FEV1/FVC of asthmatic and COPD subjects a Spearman’s correlation analysis was performed.

## Results

### Clinical characteristics of study participants

The detailed clinical characteristics of the two groups of participants recruited in this study, including demographic characteristics, smoking status and spirometry function, are shown in Table 1.

Differences between patients with COPD and those with asthma were statistically significant in terms of age (*p* value < 0.001), FEV1% (*p* value < 0.0001), Forced Vital Capacity percentage predicted (FVC%) and FEV1/FVC (*p* value < 0.01). Specifically, the COPD patient group exhibited a higher average age and lower FEV1 and FVC% values compared to the asthmatic group. Furthermore, the COPD group comprised 6 current smokers, 15 ex-smokers, and 1 never-smoker. Conversely, among the asthmatic participants, 1 individual was a current smoker, 4 were ex-smokers, and 10 had never smoked. In terms of gender distribution, the majority of COPD patients were male (20 out of 22), while among the 15 asthmatic patients, 5 were male, and 10 were female.

### Integrated metabolomics and lipidomics analysis revealed differences between asthmatic and COPD patients

In the present investigation, an untargeted integrated metabolomics and lipidomics analysis by UHPLC-MS was performed, in order to investigate the differences in induced sputum metabolomics and lipidomics patterns between asthmatic and COPD patients.

Metabolomics and lipidomics data from the two analyzed groups of patients, obtained by UHPLC-MS, were compared using multivariate statistical approaches, including PLS-DA and OPLS-DA. These multivariate analyses were used to gain meaningful information from the complex metabolomic datasets, reducing data dimensionality and obtaining a visual differentiation between asthmatic and COPD patients.

**Fig. 1:**
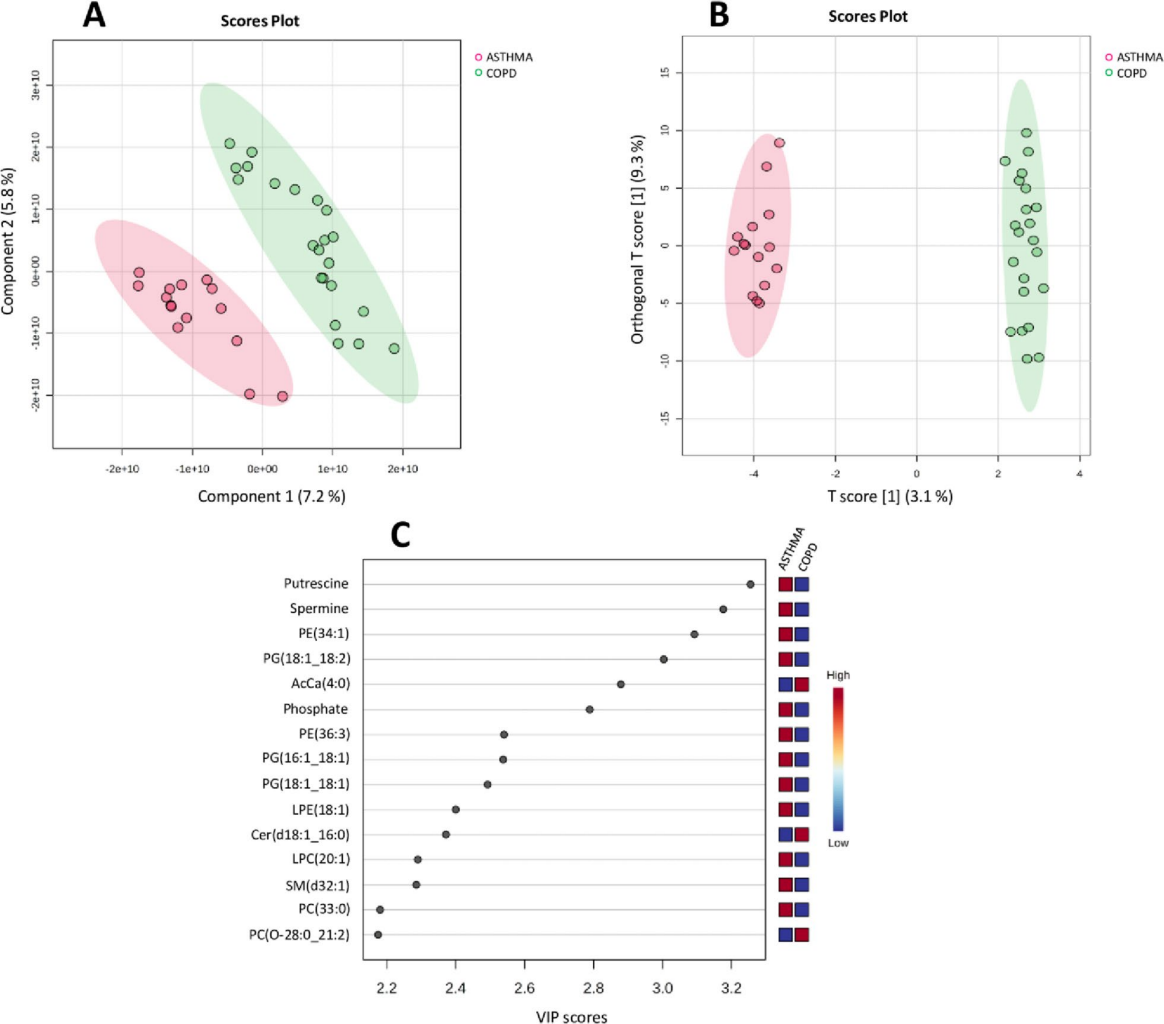
Metabolic and lipidomic data analysis of asthmatic and COPD patients using multivariate statistical methods. **A** The score plot generated by Partial Least Squares Discriminant Analysis (PLS-DA) and **B** the score plot produced by Orthogonal Partial Least Squares Discriminant Analysis (OPLS-DA) visually demonstrates a clear distinction in the metabolome and lipidome profiles between asthmatic and COPD patients. **C** The VIP score plot, generated using PLS-DA, highlights the top 15 metabolites and lipid compounds that exhibited differences between asthmatic and COPD patients. Colored boxes on the right side of the plot indicate the relative concentration of the corresponding compounds, with blue representing low concentration and red indicating high concentration. These PLS-DA, OPLS-DA, and VIP score plots were generated using the Metaboanalyst software

As shown in Fig. 1, a well-defined separation of groups was obtained by both the PLS-DA (Fig. 1A) and OPLS-DA (Fig. 1B) models. All COPD IS samples (green dots) were clustered on the right, whereas all asthma induced sputum samples (red dots) were clustered on the left, and no overlap was observed (Fig. 1A, B). This result suggested the presence of noteworthy differences in the IS metabolomic and lipidomic profiles between individuals with COPD and those suffering from asthma.

Figure 1C shows the Variable Importance in the Projection (VIP) plot that was used to identify key metabolites or lipids contributing to the distinction between the two groups. VIP plot was generated from the PLS-DA model and shows the top 15 compounds with a VIP score > 1, which are ranked based on their discriminatory power. Considering that variables with a VIP score > 1 are interpreted as being highly influential, principal compounds considered more important to the strength of segregation were Putrescine, Spermine, Phosphatidylethanolamine (PE) (34:1), Phosphatidylglycerol (PG) (18:1;18:2), AcCa(4:0), Phosphate, PE(36:3), PG(16:1;18:1), PG(18:1;18:1), Lysophosphatidylethanolamine (LPE) (18:1), Ceramide (Cer) (d18:1;16:0), Lysophosphatidylcholine (LPC) (20:1), Sphingomyelin (SM) (d32:1), Phosphatidylcholine (PC) (33:0), PC(O-28:0;21:2).

**Fig. 2:**
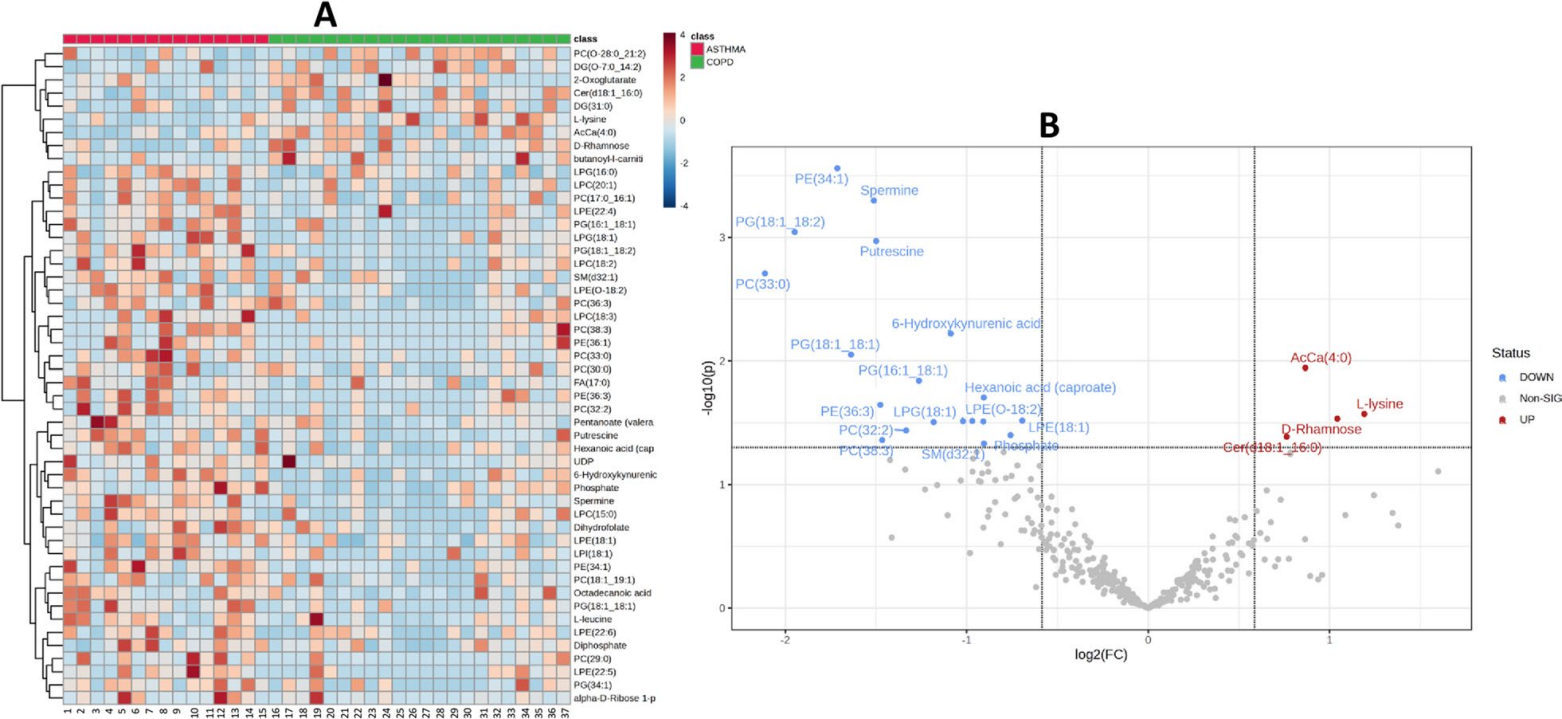
Variations in metabolite and lipid levels between asthmatic and COPD patients. **A** The heatmap illustrates the relative abundances of the top 50 metabolites and lipids in asthmatic compared to COPD patients. Red indicates an increase in abundance, while blue a decrease. The columns and rows in the heatmap correspond to the experimental samples and the compounds, respectively. **B** The volcano plot highlights metabolites and lipid compounds that exhibit significant differences between the two groups, based on fold change (COPD/ASTHMA) and *p*-value. Specifically, blue dots (Fold Change < 0.67; *p*-value < 0.05) or red dots (Fold Change > 1.5; *p*-value < 0.05) indicate significantly downregulated or upregulated metabolites, respectively. Gray dots represent compounds that did not show significant differences. Both the heatmap and volcano plot were generated using the Metaboanalyst software

Variations in metabolite and lipid levels between asthmatic and COPD patients are shown in Fig. 2. In particular, a heatmap was used to display the relative abundances of the top 50 significant metabolites and lipids (Fig. 2A), represented with color intensity, between asthmatic and COPD individuals. In addition, by combining results from fold change (> 1.5 or < 0.67) (COPD/ASTHMA), considered significant cutoff values, and FDR-corrected *p*-values < 0.05, volcano plot analysis was conducted displaying metabolites and lipid compounds that significantly contributed to the distinction between the two groups (Fig. 2B).

**Fig. 3:**
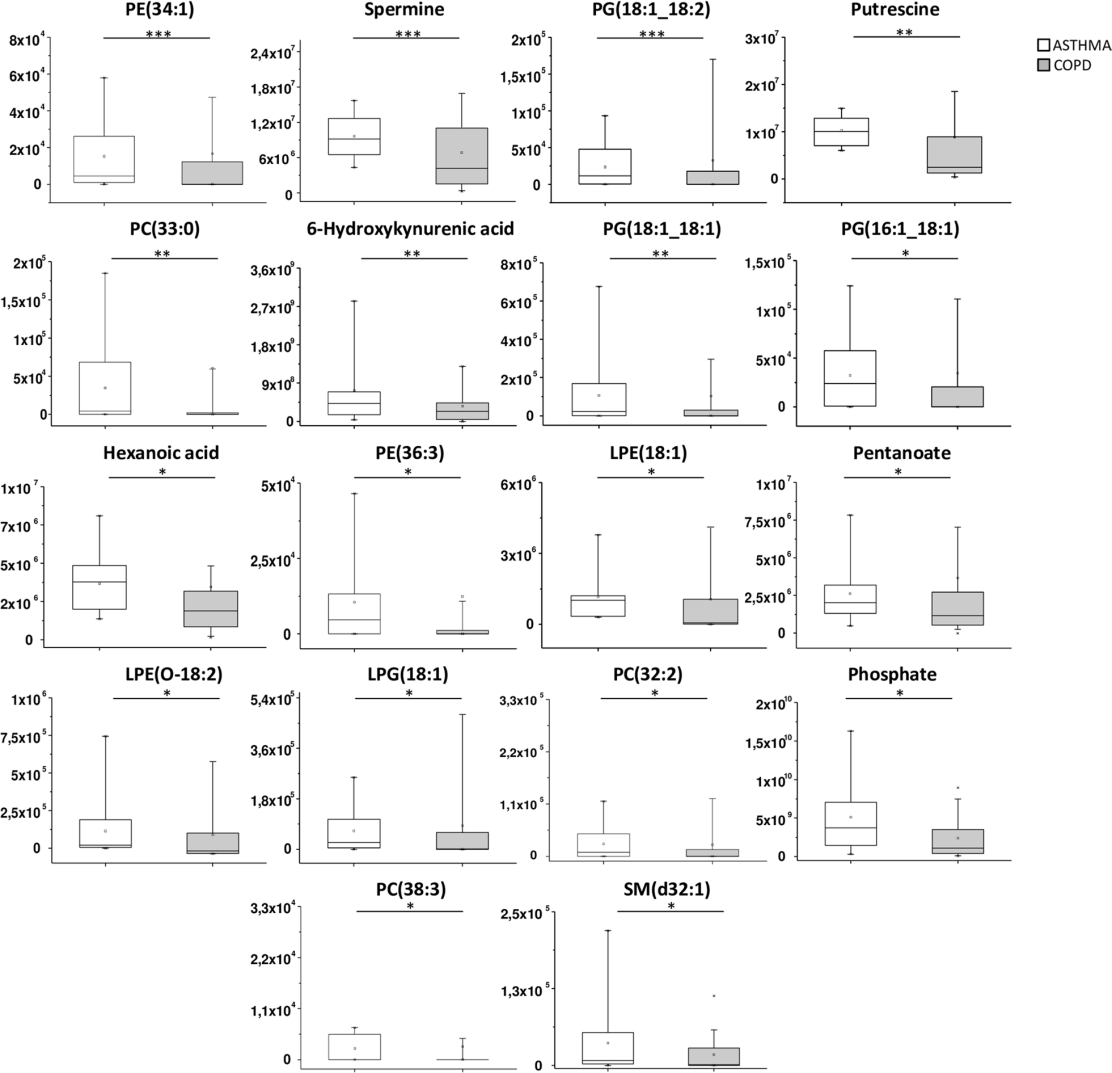
Box plot analysis of main discriminant compounds. Box plot of the peak intensities for the 18 statistically downregulated compounds in COPD patients compared to asthmatics. The p-values were calculated by Student t-test and the asterisks show the level of significance between the two groups; * *p*-values < 0.05, ** *p*-values < 0.01, *** *p*-values < 0.001. Box plot analysis was performed by OriginLab^®^ software

**Fig. 4:**
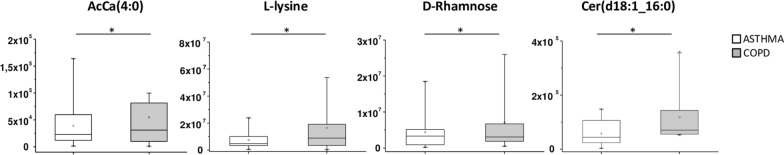
Box plot analysis of main discriminant compounds. Box plot of the peak intensities for the four statistically upregulated compounds in COPD patients compared to asthmatics. The p-values were calculated by Student t-test and the asterisks show the level of significance between the two groups; * *p*-values < 0.05. Box plot analysis was performed by OriginLab^®^ software

Subsequentially, combining results from VIP plot (VIP score > 1) and Volcano plot analysis (*p* values < 0.05 by Student t-test and fold change > 1.5 or < 0.67) 22 statistically significant compounds (including metabolites and lipids) were obtained as shown in the Table 2. In particular, 18 compounds showed a decreased concentration and 4 showed an increased concentration in COPD when compared to asthmatics patients. In order to more comprehensively assess the differences between the two groups, a box plot analysis of peak intensities (autoscale normalized, i.e., mean-centered and divided by the standard deviation of each variable) for each significant compound was also performed and results are displayed in Figs. 3 and 4.

**Table 2:** List of differential metabolites and lipids between asthmatic and COPD patients

Compound	*m/z*	Rt (s)	VIP-score	Fold change^a^	*P* value	Metabolic pathway	Class
PE (34:1)	718.5381	2.77	3.093	0.3050	0.00027	Glycerophospholipids metabolism	GPE
Spermine	203.2230	0.61	3.176	0.3506	0.0005	Arginine and proline metabolism	Polyamines
PG (18:1;18:2)	771.5182	3.11	3.003	0.2591	0.0009	Glycerophospholipids metabolism	GPG
Putrescine	89.1075	0.63	3.255	0.3538	0.001	Arginine and proline metabolism,	Polyamines
PC (33:0)	748.5851	3.01	2.181	0.2313	0.002	Glycerophospholipids metabolism	GPC
6-Hydroxykynurenic acid	206.0446	1.80	2.055	0.4704	0.006	Tryptophan metabolism	organic acid
PG (18:1;18:1)	792.5749	2.74	2.493	0.3214	0.009	Glycerophospholipids metabolism	GPG
AcCa (4:0) (butyryl-l-carnitine)	232.1543	0.25	2.879	1.8228	0.011	Energy metabolism	Fatty acyl carnitines
PG (16:1;18:1)	745.5025	3.09	2.538	0.4165	0.014	Glycerophospholipids metabolism	GPG
Hexanoic acid (caproate)	115.0765	1.82	1.517	0.5338	0.02	Fatty Acid Biosynthesis	Fatty Acids and Conjugates
PE (36:3)	742.5381	2.86	2.541	0.3594	0.02	Glycerophospholipids metabolism	GPE
l-Lysine	147.1128	0.60	2.138	2.2831	0.03	Amino acid metabolism biotin metabolism	Amino acids
d-Rhamnose	163.0612	0.67	1.630	2.0597	0.03	ABC transporters	Deoxy sugars
LPE (18:1)	480.3085	1.76	2.400	0.6184	0.03	Glycerophospholipids metabolism	GPE
Pentanoate (valerate)	101.0608	0.72	1.400	0.5109	0.03	Fatty acid biosynthesis	Fatty Acids and Conjugates
LPE (O-18:2)	462.2990	2.52	1.943	0.5329	0.03	Glycerophospholipids metabolism	GPE
LPG (18:1)	509.2885	2.22	2.149	0.4407	0.03	Glycerophospholipids metabolism	GPG
PC (32:2)	730.5381	2.76	1.986	0.3967	0.04	Glycerophospholipids metabolism	GPC
Phosphate	96.9696	0.62	2.789	0.5913	0.04	Aspartate metabolism	Inorganic compounds
Cer (d18:1;16:0)	520.5088	3.03	2.372	1.6972	0.04	Sphingolipid metabolism	Cer
PC (38:3)	812.6163	3.05	1.817	0.3620	0.04	Glycerophospholipids metabolism	GPC
SM (d32:1)	675.5435	2.65	2.286	0.5341	0.046	Sphingolipid metabolism	SM

^a^Fold change value refers to the “COPD *versus* ASTHMA” change values

*m/z* mass-to-charge ratio, *Rt* retention time, *VIP* variance importance for projection, *PE* Phosphatidylethanolamine, *GPE* glycerophosphoethanolamines, *LPE* Lysophosphatidylethanolamine, *LPG* Lysophosphatidylglycerol, *GPG* glycerylphosphorylglycerols, *PC* Phosphatidylcholine, *GPC* glycerophosphatidylcholine, *Cer* ceramides, *SM* sphingomyelins

Table 2 also lists the corresponding pathways that each identified compound was involved in, based on the knowledge derived from Kyoto Encyclopedia of Genes and Genomes (KEGG) pathway database. The differentially expressed compounds found between asthmatic and COPD patients belonged to Glycerophospholipid and sphingolipid metabolism, Arginine and Proline metabolism, Tryptophan metabolism, Energy metabolism, FA biosynthesis, ABC transporters and Biotin metabolism (Table 2).

### Validating the statistically significant compounds as potential biomarkers of differentiation between asthma and COPD by ROC analysis

**Fig. 5:**
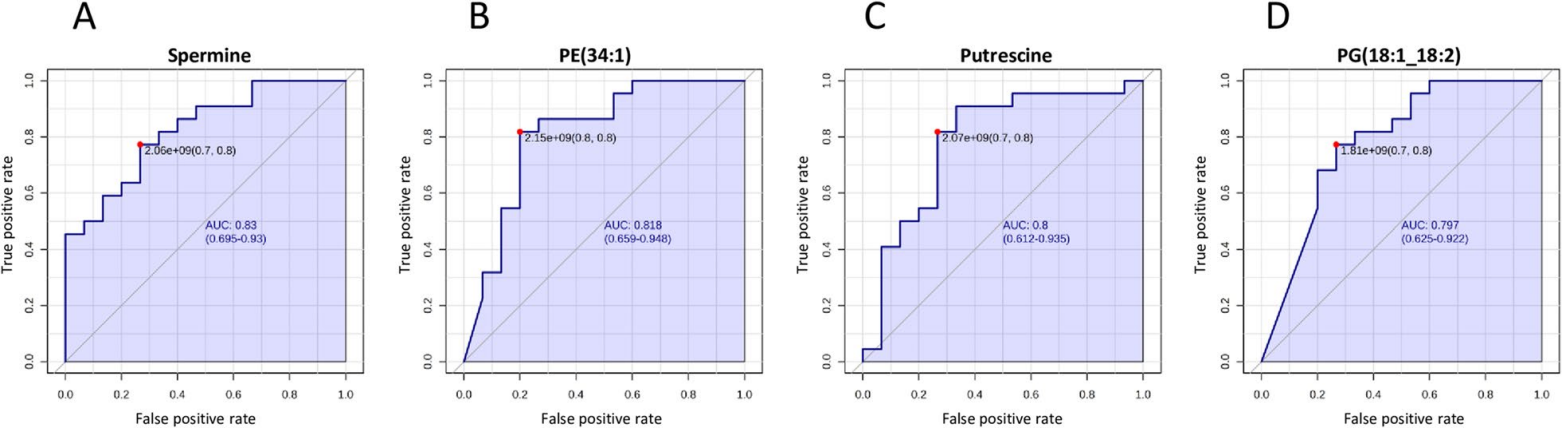
Roc curve analysis of statistically significant compounds. **A**–**D** ROC curves were constructed for compounds that exhibited the most effective diagnostic performance and a good ability to distinguish between these two groups, as measured by an Area under the Curve (AUC) around 0.8. ROC curves were acquired by Metaboanalyst software

**Fig. 6:**
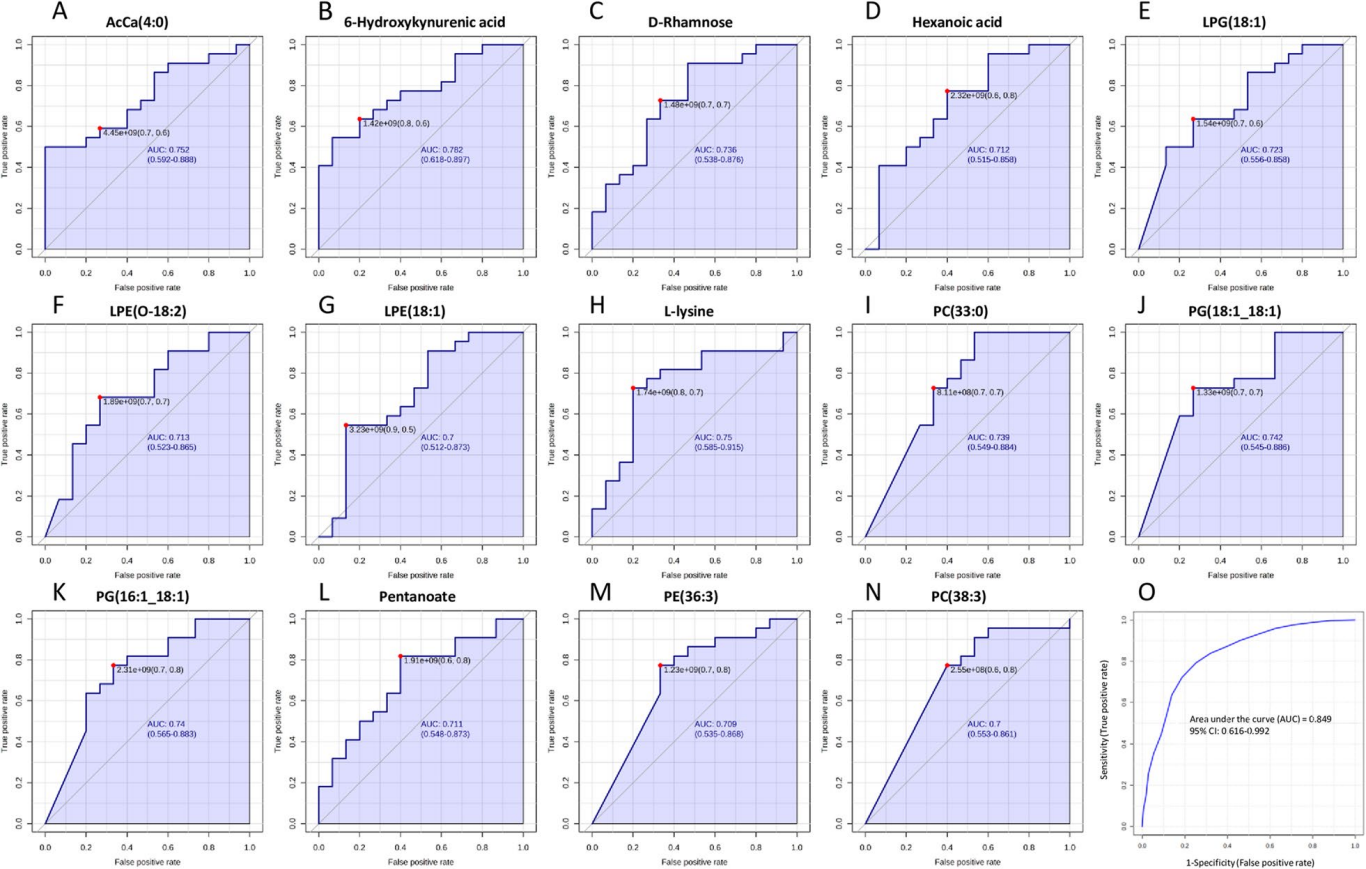
Roc curve analysis of statistically significant compounds. **A**–**N** ROC curves constructed for compounds that exhibited the most effective diagnostic performance and a moderate ability to distinguish between these two groups, as measured by an Area under the Curve (AUC) around or greater than 0.7. **O** Cumulative ROC curve constructed by combining all compounds that showed good/ moderate diagnostic capability. ROC and cumulative ROC curves were acquired by Metaboanalyst software

The differentially expressed metabolites and lipids were examined by ROC curves, aimed at evaluating the diagnostic performances of potential biomarkers, including sensitivity and specificity (Figs. 5 and 6). The area under the ROC curve (AUC) was used to detect the accuracy and efficiency of this method in distinguishing asthma and COPD patients. Our data revealed that, among all the differentially expressed compounds, 18 exhibited the highest AUC (AUC > 0.7). In particular, PE (34:1), Spermine, Putrescine and PG(18:1;18:2) had AUC values around 0.8, indicating a good discriminating capability between asthmatic and COPD patients (Fig. 5). Other compounds, including AcCa (4:0), 6-Hydroxykynurenic acid, D-Rhamnose, Hexanoic acid, lysophosphatidylglycerol (LPG) (18:1), LPE (O-18:2), LPE (18:1), L-lysine, PC (33:0), PG (18:1;18:1), PG (16:1;18:1), Pentanoate, PE (36:3) and PC (38:3) exhibited AUC values around or greater than 0.7, showing moderate abilities in the differentiation between the two group (Fig. 6A–N). Subsequently, a cumulative ROC curve was created by combining the compounds previously mentioned. This cumulative ROC curve demonstrated an AUC of 0.849 and showed a strong sensitivity (0.992) and a reasonable specificity (0.616) as depicted in Fig. 6O. This combination of potential biomarkers proved to be a valuable tool that could well distinguish between asthmatic and COPD individuals.

### Associations between IS metabolites and lipids and lung functions by spearman correlation analysis

Lung functions FEV_1_ and FVC were significantly lower in the COPD compared to asthma group showing a *p* value < 0.0001 and *p* value < 0.01 respectively (Table 1).

FEV1/FVC ratio was significantly lower in the COPD compared to asthma group (*p* value < 0.01).

**Fig. 7:**
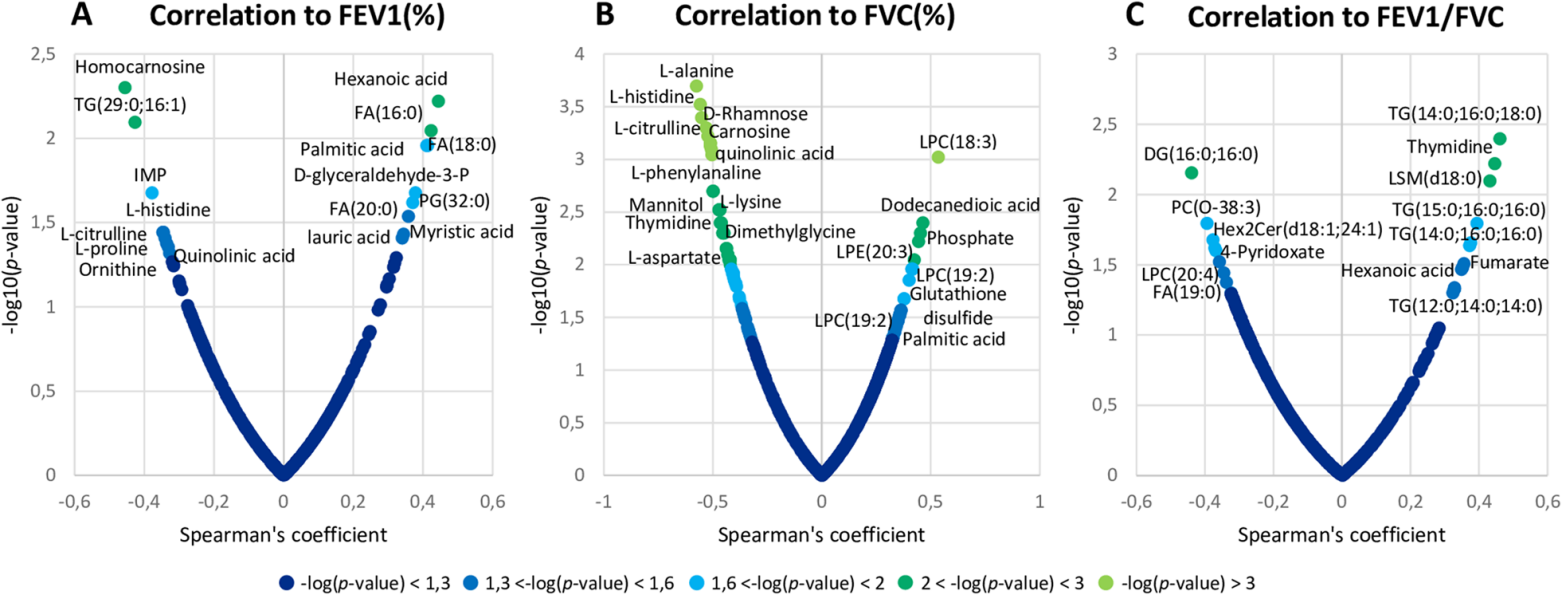
Spearman correlation analysis between compounds and lung function parameters. Each panel shows the most significant metabolites and lipids correlated to FEV1(%) (**A**), FVC(%) (**B**) and FEV1/FVC (**C**). The x-axis indicates the Spearman’s correlation coefficient and the y-axis indicates the significance of the correlation (− log_10_ of the *p*-value)

Metabolites and lipids compounds were correlated with lung function parameters (FEV1, FVC, FEV1/FVC) by Spearman correlation analysis (Fig. 7 and Additional file 1: Table S1).

Figure 7 shows the most significant compounds found to be correlated with FEV1, FVC and FEV1/FVC. In particular, FEV1(%) correlated positively, especially with Hexanoic acid, some fatty acids (FA) and PG species, Palmitic, Lauric and Myristic acid, and negatively with some triglycerides (TG) species, homocarnosine, inosine monophosphate (IMP), histidine, proline, citrulline, ornithine and quinolinic acid (Fig. 7A). FVC (%) showed a positive correlation with some LPE, LPC species, Palmitic acid, phosphate and glutathione disulfide. On the contrary, D-Rhamnose, carnosine, quinolinic acid, mannitol, thymidine and some amino acids including lysine, alanine, histidine, phenylalanine, aspartate correlated negatively with FVC (%) (Fig. 7B). Finally, FEV1/FVC exhibited a positive correlation with some TG, Lysospingomyelin (LSM) species, thymidine, fumarate and hexanoic acid, while a negative correlation was observed for some Diacylglycerols (DG), PC, LPC, FA species and 4-Pyridoxate (Fig. 7C).

## Discussion

Asthma and COPD both originate from complex interactions between environmental agents and genetic factors. In COPD, mainly caused by tobacco smoke and airborne pollutants, airflow limitation is usually poorly reversible and persistently progressive. In asthma, bronchial obstruction is extremely variable and often reversible, as it is mainly due to the pathogenic actions of respiratory viruses and aeroallergens [12, 27]. A common feature shared by asthma and COPD is their remarkable heterogeneity, expressed as multiple phenotypes characterized by different clinical, functional, and pathobiologic patterns [28, 29].

IS supplies information about both inflammatory cells and mediators present in the airways, potentially important for phenotypic characterization of patients with chronic respiratory disorders, such as COPD and asthma [11, 12]. It is well accepted that recognition of specific metabolic profiles in COPD and asthma may improve patient management strategies, including diagnosis, offering new potential targets for future tailored therapy thus providing higher patients’ probabilities for survival [30, 31]. The present investigation provides a comprehensive comparative view of the metabolic and lipidomic profile of IS samples in asthmatic and COPD patients, offering not only new insights for investigation into the mechanism of disease and identification of novel diagnostic biomarkers, but also supplying additional information to complement previous research on asthma and COPD. Until now, only a limited number of reports analyzed IS, focusing on metabolomics and lipidomics differences between asthmatic and healthy individuals, or between COPD patients and controls, never directly comparing asthma and COPD patients [9, 32, 33]. In the majority of cases, the comparison of metabolomic and lipidomic profiles between asthma and COPD has been performed using other types of bodily fluids, such as serum and urine, which do not accurately reflect the lung's specific pathophysiological state [19–21]. Only one study has been recently reported, in which metabolomics and lipidomics analyses were performed on a proximal but invasive sample such as cultured bronchial epithelial cells (BECs) comparing healthy individuals to patients with severe asthma and, for comparison, patients with COPD [34]. Other reports used exhaled breath condensate (EBC) samples to distinguish between the metabolome of these two diseases [35, 36]. However, this specific sample may contain low concentrations of biomarkers due to dilution by the water vapor of exhaled air and is difficult to normalize [33].

Concerning asthma metabolomics, it is well established that metabolomic biosignatures in EBC, urine, and blood could distinguish asthma and asthma phenotypes [10]. Many studies found a significant alteration of lipid metabolism components such as PE, PG and SM in asthma [37–39]. Interestingly, in a sputum metabolomics study, which compared 20 asthmatic patients against 15 healthy subjects, glycolysis/gluconeogenesis, glycerophospholipid, and inositolphosphate metabolism pathways associated with asthma [40]. It is widely accepted that glycerophospholipid metabolism was the most significantly perturbed pathway in experimental allergic asthma [40, 41].

Metabolomics and lipidomics studies related to COPD in different kinds of samples have shown that the alteration of lipid and amino acid metabolism, energy production pathways, the imbalance of oxidations and antioxidations oxidative stress and protein malnutrition may lead to local and systemic inflammation and, may contribute to the development and progression of COPD [9, 42].

**Fig. 8:**
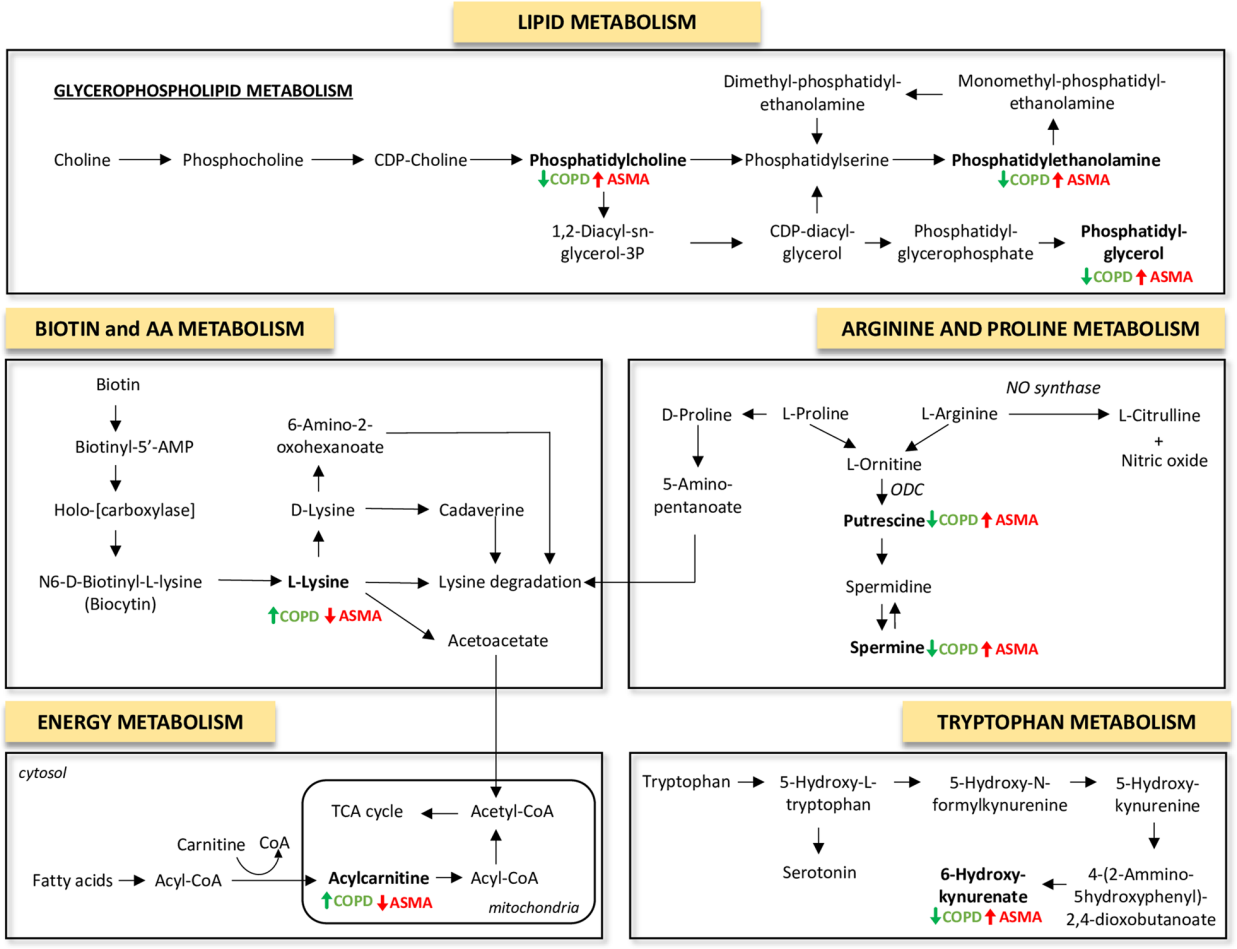
Main metabolic pathways and related analytes with significant variation of their levels in the induced sputum of COPD and asthmatic patients

In line with reported studies, our dataset has highlighted the alteration of many of the metabolic pathways (Table 2 and Fig. 8) found in previous studies. To the best of our knowledge, this is the first UHPLC-MS based approach that screens and compares the metabolomic and lipidomic profile between asthma and COPD patients in IS samples. The use of our integrated and untargeted metabolomics/lipidomics approach by UHPLC-MS has shown significant differences and alterations in specific metabolic and lipid pathways prominently associated with asthma or COPD (Fig. 8). In particular, a total of 22 compounds, including 14 lipids species and 8 metabolites, were identified to be significantly different in the IS from the two groups (Fig. 3 and Fig. 4) and well differentiate asthmatic from COPD patients (Fig. 5 and Fig. 6). The analyzed data seem to strongly confirm that asthmatic patients show a typical IS metabolomics and lipidomics profile distinct from that found in COPD subjects. Levels of lipid metabolic intermediates such as PE and PG were found significantly increased in IS of asthmatic in comparison to COPD patients (Fig. 3). Interestingly, a significant dysregulation in arginine and proline metabolism pathways, evidenced by a strikingly elevated concentration of two polyamines spermine and putrescine in asthmatic *vs* COPD, was found (Fig. 3). In this differential metabolic pattern, deduced by our data, the observed decreased level of AcCa (4:0) and Lysine in the IS of asthmatic compared to COPD patients (Fig. 4) was noteworthy. These findings seem to fit well with the established main features on asthma and COPD pathophysiology, as it will be discussed in the following sections. Asthma pathobiology, involving activation of both innate and adaptive immune systems that stimulate chronic airway inflammation in response to allergens and several external agents, results in altered pathways of glycerophospholipids, arginine and proline, as well as tryptophan metabolism (Fig. 8). On the other hand, in COPD, altered pathways seems more prominently related to biotin and amino acids as well as energy metabolism, indicating the importance of inflammatory-immune processes with increased catabolism and alterations in the energy production (Fig. 8).

## Dysregulated metabolites between COPD and asthmatic patients

### Aminoacid metabolism

Our results showed elevated levels of L-Lysine in IS of COPD compared to asthmatic patients (Fig. 4) and a significantly negative correlation between this metabolite and a lung function parameters (FVC%) was also found (Fig. 7 and Additional file 1:Table S1). Lysine is involved in biotin metabolism, as a product of biotin protein turnover during carboxylation reactions [43]. Importantly, it plays a pivotal role in the synthesis of carnitine, which is essential for fatty acids metabolism [44].

In accordance with our data, Khamis et al*.* found an increase of lysine levels in the urine samples of COPD compared to asthmatic patients [21]. Furthermore, other studies demonstrated that lysine was associated with worsening severity of COPD exacerbations [45, 46], suggesting a potential implication of this amino acid in this pathology.

Interestingly, lysine was dysregulated in ACO patients when compared with both asthma and COPD [47]. In particular, the mentioned study demonstrated that lysine levels are significantly lower in ACO patients as compared to asthma and COPD.

To the best of our knowledge, this is the first report to find significantly different levels of L-lysine between asthma and COPD patients in IS samples. Additionally, the negative correlation we found between this metabolite and the lung parameter FVC (%) (Fig. 7B and Additional file 1: Table S1), implies that high levels of lysine are associated with a decrease in FVC (%). Diminished FVC (%) values are typically observed in individuals diagnosed with COPD (Table 1). Consequently, our findings are consistent with the increased lysine levels observed in COPD patients (Fig. 4), suggesting a strong association of this amino acid with the development of COPD.

It is tempting to speculate that the lower levels of lysine found in asthmatic patients might be due to the higher metabolic demand in these subjects to generate acetyl-CoA. Concomitantly, acylcarnitine levels result lower in asthmatic compared to COPD patients (Figs. 4 and 8) because needed to supply the production of acetyl-CoA (Fig. 8). Therefore, a convergent regulatory synergistic mechanism could be involved in the production of acetyl-CoA providing its involvement in the TCA cycle (Fig. 8). Obviously, the significance of this finding warrants further investigations.

### Polyamines biosynthesis

In our study, altered levels of two polyamines, spermine and putrescine, are observed between asthmatic and COPD patients (Fig. 3). As already stated, spermine and putrescine showing AUC values around 0.8 indicated a good discriminating capability to correctly estimate a random subject as asthmatic or COPD (Fig. 5).

Polyamines are naturally occurring small-sized super-cations that interact with intracellular macromolecules carrying negative charges. In mammals, polyamines are synthesized from the decarboxylation of arginine- and proline-derived ornithine by the enzyme ornithine decarboxylase (ODC) (Fig. 8) [48].

Changes in polyamine levels are associated to various pathological conditions, including asthma [49].

Polyamines have a significant role in lung physiology, as they are specifically accumulated in the epithelial cells of the lung [50].

Increased levels of polyamines have been reported in the blood of individuals with asthma and in the lungs of asthma mouse models [51, 52]. Polyamines were found to enhance the pathological aptitude of inflammatory cells, like mast cells and granulocytes, either stimulating the release of pro-inflammatory substances from these cells or extending their lifetime [53, 54].

North and colleagues detected elevated levels of putrescine and spermine in sputum samples of asthmatic patients after an allergen challenge [55]*.* They also demonstrated that endogenous concentrations of these polyamines were significantly increased in the lungs of a murine model of ovalbumin-induced allergic inflammation. In particular, their data showed that, when relevant concentrations of spermine were administered into the airways, airway responsiveness was enhanced both in naïve mice and in mice with allergic inflammation [55]. It is worth of note that an increased biosynthesis of polyamines exacerbates lung diseases; in fact, the synthetic pathway of these species competes for L-arginine at the expenses of NO production, leading in this way to bronchoconstriction (Fig. 8). By the way, exhaled NO is produced from arginine and several studies highlight the association with asthma and asthma severity [56, 57].

Our study demonstrated that spermine and putrescine were some of the most important metabolites for group segregation as a statistically significant increase of these two polyamines was observed in asthmatic compared to COPD patients (Figs. 3 and 5). Specifically, the asthma group showed higher levels of spermine and putrescine, confirming previous literature data.

Our results are the first to demonstrate different levels of these polyamines between asthma and COPD groups in IS samples. These findings might suggest that spermine and putrescine are strongly associated with airway hyperresponsiveness that typically occurs in asthmatic individuals [27]. Given the previous observation, it is tempting to hypothesize that their synthetic pathway is correlated to a lower level of arginine (see Fig. 8). Low arginine levels are associated with impaired smooth muscle relaxation [58]. In line with this, we observed a slight decrease in arginine levels in asthmatic subjects compared to COPD patients (Additional file 2: Fig. S1 and Additional file 3: Table S2). A similar trend of arginine levels was also found in urine samples in a metabolomic comparative analysis between asthma and COPD subjects [20]. Interestingly, cytrulline, which is formed as by-product together with NO from arginine (Fig. 8), was found among the seven top metabolite negatively associated with FVC (-log (p value) > 3) (Fig. 7 and Additional file 1: Table S1) in line with the above mentioned hypothesis.

### Fatty acid biosynthesis related compounds

A statistically significant decrease of Hexanoic acid was found in the COPD group compared to the asthmatic one (Fig. 3). A significantly positive correlation between Hexanoic acid and lung function parameters (FEV1% and FEV1/FVC) was also observed (Fig. 8 and Table S1).

Hexanoic acid (or caproic acid) is a short-chain fatty acid (SCFAs) produced through bacterial fermentation of dietary fiber within the gut [59, 60]. Increasing evidence indicates the existence of a metabolic and immune connection between the gut and the respiratory system [61, 62] and suggests SCFAs as the key components in establishing this connection. The association between lung and gut was confirmed by the identification of SCFAs in the sputum of patients with cystic fibrosis [63]. Interestingly, SCFAs: (i) contribute to the regulation of immune-metabolic tone in the lung [64]; (ii) to have anti-inflammatory effects in asthma by downregulating allergic immune response and inflammasome pathway [65]; (iii) participate in maintaining the integrity of damaged airway epithelium by upregulating the expression of dense contact proteins such as ZO-1 [66]. This is particularly significant considering the existence of dysfunction in airway epithelial barrier and tight cell contacts associated with smoking and COPD [67]. Consequently, SCFAs might have a potential clinical significance in repairing airways barrier function [66].

Interestingly, a in study by Li et al*.* the level of seven SCFAs (including caproic acid) in the stool samples of COPD patients and healthy individuals was compared, showing a lower level of total SCFAs in the COPD group compared to healthy [68]. Although in this study the authors analyzed fecal samples, their findings may support the data from our study on IS samples, given the existence of a connection between the gut and the lungs.

### Tryptophan metabolism related compound

6-Hydroxykynurenic acid was another metabolite found to be statistically significantly decreased in COPD compared to the asthmatic group (Fig. 3). This metabolite is a derivative of kynurenic acid that takes part to the tryptophan metabolism.

Tryptophan is an essential amino acid with important functions including cellular protein synthesis and the formation of the cytoskeleton [69] and it can be metabolized via different pathways in several cell types.

Tryptophan and its metabolites have been investigated in the context of inflammatory and respiratory diseases, revealing alterations in their levels during infections, as a consequence of the immune response [70]. In the lung, tryptophan catabolism leads to the accumulation of metabolites of the kynurenine pathway by the activity of the enzyme indoleamine-pyrrole 2,3-dioxygenase (IDO), thus resulting in immune tolerance and an anti-inflammatory effect. The induction of immune tolerance has been linked to lung cancer and HIV infection [71]. IDO, predominantly expressed in macrophages and dendritic cells, has also been shown to act as an important T cell immunomodulator [72].

Alteration in tryptophan metabolism was also observed in the sputum of COPD patients, attributed to a decrease in hydroxy-indolacetic acid levels [73], which could potentially lead to a diminished lung defense against neutrophilic inflammation and epithelial apoptosis. Ubhi and colleagues found decreased tryptophan levels in the serum of COPD patients with emphysema [74]. Noteworthy, Maneechotesuwan et al*.* observed a decrease in the activity of IDO in the sputum of COPD patients, and this reduction was associated to the severity of the disease. This finding suggests that the decline in IDO activity within sputum contributes to an environment that promotes neutrophilic inflammation [75]. Interestingly, IDO contributes to the suppression of intracellular reactive oxygen species (ROS) in acute lung allograft injury and improves the resistance of lung cells to oxidative stress [76]. Therefore, our data might suggest a decrease of IDO activity within IS of COPD compared to the asthmatic patients, which resulted in the observed lower levels of 6-hydroxykynurenic acid in COPD compared to asthma. The hypothesized decrease of IDO activity in COPD might also suggest a concomitant mechanism associated to increased expression of ROS resulting in major oxidative stress in COPD.

It is well known, that tryptophan and its metabolites can interact and/or generate ROS. This is an important topic, as alterations in the kynurenine pathway metabolites, oxidative stress, energetic deficit, cell death and inflammation may converge in a network of pathogenic mechanism [77]. Up to date, this pathway has attracted an intense research interest in the fields of aging and brain diseases [78, 79]. The identification of this novel metabolite in IS by our approach may open new avenues for exploring the involvement of this pathway also in chronic inflammatory lung diseases and this topic surely warrants future investigations.

### Other compounds

In this investigation, a significantly altered level of phosphate was found between COPD and asthmatic patients (Fig. 3).

In its ionized form, inorganic phosphate, is involved in common cellular processes, such as regulating energy metabolism, promoting bone mineralization, facilitating membrane transport and enabling intracellular signaling [80]. Furthermore, respiratory muscle contractility, electrolyte transport and the response to inflammation can be affected by phosphate [81]. Recent investigations showed that alteration in phosphate levels can promote adverse outcomes in many diseases [82, 83]. Few studies have suggested that there is a correlation between serum phosphate levels and the severity of COPD. For instance, Stroda et al*.* showed that serum phosphate levels are lower in COPD patients compared to controls [84]. Farah and colleagues also suggested that hypophosphatemia was related to an increase of COPD exacerbation, duration of hospitalization and mortality rate [85]. On the contrary, other studies revealed that high level of serum phosphate was associated with mortality in COPD patients [81, 86].

According to our current knowledge, the present study is the first to show a significant decrease of phosphate levels in the IS of COPD compared to asthmatic patients; additionally, phosphate shows a positive correlation with FVC (%) (Figs. 3, 7B and Additional file 1: Table S1), suggesting a potential application of this metabolite in the differentiation between these two lung conditions.

Another metabolite able to discriminate COPD from asthmatic patients was d-Rhamnose.

Rhamnose is a naturally occurring deoxy sugar. Its D form (d-Rhamnose) is a component of cell surface polysaccharides in some species of bacteria such as Pseudomonas aeruginosa [87], a pathogenic bacteria that frequently cause infections, especially in individuals with compromised immune systems. These bacteria are a primary cause of lung infections in patients with cystic fibrosis and are linked to a significant rate of illness [88]. Pseudomonas aeruginosa has also been isolated from bronchial secretions in patients with COPD [89, 90] and asthma [91]. It has also been correlated with a high frequency of exacerbation, increased respiratory symptoms, rapidly worsening of lung function, poor quality of life and increased mortality [92].

To the best of our knowledge, our study is the first to identify d-Rhamnose as a potential discriminative biomarker between asthma and COPD in IS. Therefore, the upregulation of this metabolite found in COPD group might suggest a higher prevalence of Pseudomonas aeruginosa infection in COPD patients compared to asthmatic. This elevated level of D-Rhamnose in COPD patients in comparison to asthma is confirmed by the strong negative correlation between the metabolite and FVC(%) observed in our investigation (Fig. 7B and Additional file 1: Table S1). Specifically, as d-Rhamnose levels increase, FVC (%) values decrease. Notably, diminished values of this lung parameter were consistently observed in COPD patients (Table 1). Several studies support the hypothesis that bacterial colonization of the airways induces inflammation and impairs lung function (FEV1) [93]. Therefore, the metabolomics approach performed in this study might support future research to more accurately characterize the immune-phenotyping airway microbiota of both asthma and COPD, which in turn, could address the development of novel targeted immuno-therapeutics.

### Dysregulated lipid species between COPD and asthmatic patients

#### Glycerophospholipid related metabolism

Glycerophospholipids play significant roles in cell membranes as major constituents, serving as storage materials for bioactive compounds. Recent research has highlighted the involvement of glycerophospholipids in the development of lung infections [94], asthma and COPD [19, 46, 95, 96]. These lipids are also constituents of lung surfactants, which can be compromised by smoke exposure [97]. They are released by airway epithelial cells into the alveoli to minimize alveolar surface tension and prevent the invasion of pathogens.

In our investigation, reduced levels of different PC [PC (33:0), PC (32:2), PC (38:3)], PE [PE (34:1), PE (36:3)] and LPE [LPE (18:1), LPE (O-18:2)] species were found in COPD group compared to asthma (Fig. 3).

In a study by Cruickshank-Quinn et al*.* based on an integrated transcriptome/metabolome analysis, the authors demonstrated a gradual decrease of some glycerophospholipids species, including PC in association with declining respiratory function in blood samples [46]. Kilk and colleagues found reduced level of some PCs, including PC (38:3), in the EBC of patients with COPD compared to control individuals [98]. Halper-Stromberg et al*.* revealed significant reductions in PE, PC in the bronchoalveolar lavage fluid (BALF) of COPD patients.

In the study by Gai et al*.*, a decrease level of LPE was found in acute exacerbation stage of COPD patients and LPE (18:1) was among these lipids [97].

These findings suggest a correlation between LPE, PE, PC and COPD. Alteration of lipid metabolism associated to alveolar surfactants in COPD patients [46] might offer a promising avenue for future studies aimed at restoring alveolar surfactants and potentially treating COPD.

Dysregulation of glycerophospholipids metabolism was also found in patients with asthma. For instance, Jiang and colleagues observed a higher level in plasma of some PEs species including PE(38:1), with structural similarity to PE(34:1) (Fig. 3), in asthmatic compared to healthy controls and a positive correlation with the severity of the condition [37]. In another study, increased levels of LPC have been reported in the BALF of patients with asthma [99]. Ravi et al*.*, also found upregulation of PCs, LPCs and LPEs in the bronchial epithelial cells from patients with severe asthma compared with controls [34].

In the present work, we found that various phosphatidylglycerol (PG), including PG (18:1;18:2), PG (18:1;18:1), PG (16:1;18:1), and LPG, including LPG (18:1), were reduced in COPD group compared to asthmatic. PGs play a role as pulmonary surfactants, which are crucial for supporting efficient gas exchange and maintaining optimal respiratory function. Additionally, they are actively involved in organizing the surfactant complex, modulating the immune response, and contributing to the body's defense mechanisms [100].

In individuals with COPD, it has been observed that surface lipid levels decreased by 60%, and there was a notable reduction in PG levels in the BALF [101]. In a work by Liu et al*.*, PGs levels were decreased in serum of COPD patients with exacerbation compared to healthy and this reduction could potentially be attributed to airway damage resulting from insufficient surfactant function [100].

In our study a positive correlation was found between IS levels of LPC (18:3) and FVC% (Fig. 7 and Additional file 1: Table S1). As expected, significantly diminished levels of several GLP in subjects with COPD in comparison to asthmatic can lead to a more reduced lung functions in COPD patients.

To the best of our knowledge, although several above-mentioned investigations have already pointed out the alterations of glycerophospholipids metabolism in asthma and COPD patients compared to healthy individuals, only two studies including the present and the study by Ravi et al*.* [34], have pointed out on glycerophospholipids dysregulations in more specific and less systemic specimen. Targeted comparative lipidomics analyses on IS samples might drive future investigations to identify the exact role of GPL in chronic lung diseases as this sampling methods contains less confounding factors in comparison to other systemic bio-fluids.

#### Sphingolipid related metabolism

Sphingolipids play a crucial role as structural elements within cellular membranes [102]. Dysregulation in the sphingolipid metabolism has been observed in lung diseases [103, 104]. In particular, alterations in ceramide and sphingomyelin levels have been implicated in COPD. Ceramides were found to be increased in lung tissue of smokers and patients with COPD and have been associated with the induction of pulmonary vascular cell apoptosis caused by tobacco smoke [105]. Telenga and colleagues, identified significantly higher levels of 28 ceramides, including Cer(d18:1;16:0), in sputum from smokers with COPD compared to smokers without COPD [95].

On the other hand, low levels of SM have been associated with COPD severity [103].

Dysregulation in sphingomyelins and ceramides levels has also been reported in asthma. In fact, some SM and Cer species were found to be increased and decreased, respectively, in asthmatic patients compared to healthy individuals [37].

In our investigation, we found a statistically significant reduced level of SM(d32:1) and an increase in Cer(d18:1_16:0) in COPD patients compared to asthmatics. This increase in Cer and decrease in SM observed in our study could lead us to hypothesize an unbalance in production of SM starting from Cer, maybe due to a deficit in SM synthase expression in patients with COPD or alternatively to an increased degradation of SM due to an upregulation of sphingomyelinases activity.

To the best of our knowledge, our study is the first to evaluate the differential sphingolipids profiling between asthma and COPD in IS samples.

#### Energy metabolism related compounds

One of the other lipid compounds found to be differentially altered between the two analyzed groups was AcCa (4:0), or butyryl-l-carnitine. In particular, in our study it was more elevated in COPD compared to asthmatics patients. AcCa (4:0) is an acylcarnitine formed when an acyl group is transferred from coenzyme A to a molecule of L-carnitine.

L-carnitine and acylcarnitines are essential for oxidative catabolism of the fatty acids and changes in carnitine and acylcarnitine profiles can cause alteration in energy production, leading to deficiencies in fatty acid oxidation and complications in mitochondrial metabolism [106].

Several studies related to COPD have found changes in the level of acylcarnitines [107–110].

Kim et al. used LC-MS on both serum and urine samples in a study of 59 TB-related COPD patients, 70 smoking-related COPD patients, and 39 healthy controls (including never-smokers and smokers) and found differences in the acyl carnitines [109].

Similar results were shown in a COPDGene discovery cohort of 839 subjects and a SPIROMICS.

replication cohort of 446 subjects using LC-MS on serum samples [107]. The differences in carnitines in COPD Gene and SPIROMICS were more pronounced in women compared to men. In particular, in this investigation, propionylcarnitine (C3) showed a positive association with COPD status; on the contrary medium chain, decanoylcarnitine (C10), cis-4-decenoylcarnitine (C10:1), laurylcarnitine(C12) and myristoleoylcarnitine (C14:1) showed a negative correlation. Interestingly, serum 2-methylbutyrylcarnitine (C5) was found among the three top metabolite positively associated with FEV1 in a large cross-sectional study (n = 4742) of three population cohorts with 393 COPD cases [111].

Naz et al*.* observed an elevation in circulating levels of acylcarnitines in COPD, suggesting a greater energy demand which is reflected in the increased transfer of acetyl coenzyme A to the tricarboxylic acid (TCA) cycle [110]. The upregulation of the TCA cycle results in higher ATP production and the elevated levels of extracellular ATP in the airway have been associated with the development of COPD. This is due to its role in recruiting and activating inflammatory cells, thereby causing inflammation and tissue deterioration [112]. However, an elevated endobronchial ATP concentration has been also detected in models of asthma [113]. An increase of endobronchial ATP both in asthma and COPD would support the pathophysiologic similarities between these two diseases as already established from genetic investigations supporting this concept [114].

Reinke and colleagues, in a large-scale MS-based investigation of the urinary metabolome in adult patients with asthma, with the aim to delineate disease- and oral corticosteroids-associated metabolic differences in asthma, found that short-chain carnitines represented the strongest metabolic signature associated with asthma severity. Specifically, they found a decreased abundance of acetyl − carnitine (C2) and propionyl − carnitine (C3:0) in relation to disease severity which was independent of oral corticosteroids treatment [115].

In our investigation, the level of the acylcarnitine (butyryl-l-carnitine) resulted to be significant increased in COPD group compared to asthmatic (Fig. 4). This result might suggest that, in line with what was previously hypothesized [110] the increased levels of acylcarnitine (butyryl-l-carnitine) in IS of COPD subjects may possibly be associated to the higher demand of production of acetyl-CoA in request to an higher energy consumption in COPD compared to asthmatic patients. This hypothesis would fit with the concomitant increased level of Lysine in COPD subjects compared to the asthmatic as already observed previously [See section “Aminoacid metabolism” and Fig. 8), which would contribute to acetyl-CoA production via Acetoacetate (Fig. 8).

### Limitations of the study

The small group size and the fact that both asthma and COPD are multifactorial conditions characterized not only by a great heterogeneity but also by an important phenotypic complexity [116] may possibly limit the accuracy of this preliminary panel of putative diagnostic biomarkers to distinguish asthma from COPD. Future studies are required to confirm the selected pattern of metabolites and lipids in larger sample cohorts in order to increase the confidence and the statistical power of the analysis, which could provide a better understanding of different metabolism in asthma and COPD. At the same time, other novel metabolites and lipid intermediates differentiated in asthma and COPD could be more accurately revealed in a larger cohort.

Several factors may be causative of metabolome fluctuation including sex, age and smoking status. The mismatched age between Asthma and COPD patients as well as the sex and smoking status differences (Table 1) make the interpretation of the observed dysregulated metabo-lipidomics profile challenging because it is difficult distinguishing the differences related to aging, sex and tobacco exposure from those related to the specific disease. Nonetheless, in clinical practice, subjects suffering of COPD commonly include smokers and aged individuals. Therefore, it is quite challenging exploring these confounding factors also on larger cohorts. Further validation on stratified population subsets for both asthmatic and COPD subjects might be also an important goal to reach in future studies for better evaluate phenotypic variation and severity degrees also in relation to metabolomics dataset and lung functions.

The fact that no healthy or other control group have been included is another limitation of this study. Induction of sputum may cause such discomfort especially in healthy individuals with less expectorate fluids or in those with bronchiectasis with less expectorate fluids and consequently this is critical issue in heathy subjects sampling procedure. However, the highly specificity of the IS in the analyzed groups, which accurately reflects inflammatory changes at the site of tissue damage, provides more accurate dataset about both inflammatory cells and mediators present in the airways better than other fluid such as serum or urine which instead provide confounding systemic information.

## Conclusions

Despite intensive research efforts involving huge economic and human resources, molecular and cellular mechanisms underlying the pathology and differentiation between COPD and asthma are not yet well understood. In this study, with the aim to more accurately differentiate asthma and COPD, an untargeted metabolomics and lipidomics approach has been performed based on UHPLC-MS and MS/MS analysis of IS, a highly specific fluid reflecting the closest features of the respiratory tract. Therefore, the resulting molecular signatures extrapolated by this pilot study might reflect specific inflammatory and immune response specifically associated to asthma or COPD. Our comparative metabolomics analysis revealed 22 metabolites and lipids which differ in a statistically significant manner in the IS of asthmatic and COPD subjects. Our findings demonstrated a prominent group segregation for PE (34:1), PG (18:1;18:2), spermine and putrescine. Many altered metabolic pathways have been identified including glycerophospholipids, arginine and proline, biotin and aminoacids, tryptophan and energy metabolism, among those, which prominently segregated asthma and COPD. Further targeted analysis will be helpful to validate these IS biomarkers. If validated on a larger prospective cohort, the metabolite panel of biomarkers identified in the IS in the present study might suggest novel strategies to address more accurate asthma and COPD diagnosis, stratification and interventions.

### Supplementary Information

**Additional file1: Table S1.** Spearman’s correlation analysis between compounds and lung functions.

**Additional file2: Figure S1.** Heatmap illustrating the relative abundance levels of all the metabolites and lipids in asthmatic compared to COPD patients. Red indicates an increase in abundance, while blue a decrease. The columns and rows in the heatmap correspond to the experimental samples and the compounds, respectively.

**Additional file3: Table S2.** Peak intensities values of metabolites and lipids in all the asthmatic and COPD patients analyzed in this study.

## Data Availability

All data supporting the findings of this study are available within the paper and its Supplementary Information. However, the individual data of patients participating to this study are not openly available due to reasons of sensitivity and are available from the corresponding author upon reasonable request. The datasets used and/or analyzed during the current study are available from the corresponding author upon reasonable request.

## Abbreviations

ACO: Asthma-COPD overlap
AUC: Area under the curve
BALF: Bronchoalveolar lavage fluid
BECs: Bronchial epithelial cells
COPD: Chronic obstructive pulmonary disease
Cer: Ceramide
EBC: Exhaled breath condensate
FEV1%: Forced expiratory volume in 1 s percentage
FVC%: Forced vital capacity percentage predicted
GPC: Glycerophosphatidylcholine
GPE: Glycerophosphoethanolamines
GPG: Glycerylphosphorylglycerols
IDO: Indoleamine-pyrrole 2,3-dioxygenase
IS: Induced sputum
KEGG: Kyoto encyclopedia of genes and genomes
KYN: Kynurenine
LC–MS: Liquid chromatography-mass spectrometry
LPE: Lysophosphatidylethanolamine
LPG: Lysophosphatidylglycerol
MS: Mass spectrometry
OPLS-DA: Orthogonal partial least squares discriminant analysis
PC: Phosphatidylcholine
PE: Phosphatidylethanolamine
PG: Phosphatidylglycerol
PLS-DA: Partial least squares discriminant analysis
PCA: Principal component analysis
ROC: Receiver operating characteristic
SCFA: Short-chain fatty acid
SD: Standard deviation
SM: Sphingomyelin
TRP: Tryptophan
UHPLC: Ultra-high-pressure liquid chromatography
VIP: Variable importance plots
