# Publisher Correction to: An integrated metabo‑lipidomics profile of induced sputum for the identification of novel biomarkers in the differential diagnosis of asthma and COPD

**DOI:** 10.1186/s12967-024-05139-1

**Published:** 2024-04-05

**Authors:** Serena Correnti, Mariaimmacolata Preianò, Fabia Gamboni, Daniel Stephenson, Corrado Pelaia, Girolamo Pelaia, Rocco Savino, Angelo D’Alessandro, Rosa Terracciano

**Affiliations:** 1 Department of Health Sciences, Magna Graecia University, Catanzaro, 88100 Italy; 2 Department of Biochemistry and Molecular Genetics, University of Colorado Anschutz Medical Campus, Aurora, CO 80045 USA; 3 Department of Medical and Surgical Sciences, Magna Graecia University, Catanzaro, 88100 Italy; 4 Department of Experimental and Clinical Medicine, Magna Graecia University, Catanzaro, 88100 Italy

**Correction to: Journal of Translational Medicine (2024) 22:301**

10.1186/s12967-024-05100-2

Following publication of the original article [1], we have been notified on the following publisher’s mistake in not marking the author as the corresponding author.

It is now:

Rosa Terracciano^4^

It should be:

Rosa Terracciano^4*^
