# Supplementary material for: An integrated metabo-lipidomics profile of induced sputum for the identification of novel biomarkers in the differential diagnosis of asthma and COPD
Source: J Transl Med. 2024 Mar 23;22:301. doi: 10.1186/s12967-024-05100-2 (PMC10960495; doi:10.1186/s12967-024-05100-2)
Supplement: Supplementary file 1 — Additional file1: Table S1. Spearman’s correlation analysis between compounds and lung functions. [file 12967_2024_5100_MOESM1_ESM.pdf]

Table S1. Spearman’s correlation analysis between compounds and lung functions.

Compound	FEV1%		FVC%		FEV1/FVC	
	Spearman's coefficient	*p*-value	Spearman's coefficient	*p*-value	Spearman's coefficient	*p*-value
L-alanine	-0.320	ns	**-0.575**	**< 0.001**	0.073	ns
L-aspartate	-0.215	ns	**-0.421**	**0.009**	0.085	ns
L-glutamate	-0.265	ns	**-0.439**	**0.007**	0.101	ns
L-glutamine	-0.233	ns	**-0.424**	**0.009**	0.083	ns
glycine	-0.159	ns	**-0.406**	**0.013**	0.146	ns
L-histidine	**-0.346**	**0.036**	**-0.558**	**< 0.001**	0.043	ns
L-leucine	-0.248	ns	**-0.359**	**0.029**	0.054	ns
L-lysine	-0.298	ns	**-0.469**	**0.003**	0.067	ns
L-phenylalanine	-0.209	ns	**-0.499**	**0.002**	0.157	ns
L-proline	**-0.336**	**0.042**	**-0.423**	**0.009**	-0.013	ns
L-serine	-0.203	ns	**-0.421**	**0.010**	0.038	ns
L-tyrosine	-0.155	ns	**-0.432**	**0.008**	0.193	ns
L-valine	-0.067	ns	**-0.335**	**0.043**	0.242	ns
AMP	-0.097	ns	**-0.331**	**0.046**	0.149	ns
Thymidine	0.026	ns	**-0.461**	**0.004**	**0.446**	**0.006**
IMP	**-0.378**	**0.021**	**-0.370**	**0.024**	-0.087	ns
Inosine	-0.029	ns	**-0.437**	**0.007**	0.267	ns

Allantoate	-0.299	ns	**-0.471**	**0.003**	0.051	ns
(S)(+)-Allantoin	-0.248	ns	**-0.336**	**0.042**	-0.007	ns
Urate	-0.226	ns	**-0.338**	**0.041**	0.031	ns
5-6-Dihydrothymine	-0.269	ns	**-0.402**	**0.014**	0.068	ns
4-Pyridoxate	-0.158	ns	0.069	ns	**-0.358**	**0.030**
Phosphate	0.163	ns	**0.444**	**0.006**	-0.135	ns
Diphosphate	0.120	ns	**0.453**	**0.005**	-0.200	ns
D-Glyceraldehyde 3-phosphate/Glycerone phosphate	**0.378**	**0.021**	0.238	ns	0.265	ns
Pyruvate	-0.136	ns	**-0.511**	**0.001**	0.232	ns
Lactate	-0.107	ns	**-0.407**	**0.012**	0.140	ns
Maltotriose	-0.086	ns	**-0.500**	**0.002**	0.240	ns
Mannitol	-0.316	ns	**-0.468**	**0.003**	0.055	ns
D-Ribose	-0.009	ns	**-0.392**	**0.016**	0.273	ns
D-Rhamnose	-0.267	ns	**-0.523**	**<0.001**	0.121	ns
D-Arabitol	-0.276	ns	**-0.407**	**0.012**	-0.045	ns
Citrate	-0.257	ns	**-0.512**	**0.001**	0.132	ns
2-Oxoglutarate	-0.028	ns	-0.311	ns	**0.328**	**0.047**
Succinate	-0.053	ns	**-0.357**	**0.030**	0.203	ns

Fumarate	0.055	ns	-0.314	ns	**0.356**	**0.031**
2-Hydroxyglutarate/Citramalate	-0.216	ns	**-0.507**	**0.001**	0.180	ns
alpha-D-Ribose 1-phosphate	0.295	ns	-0.029	ns	**0.352**	**0.033**
Glutathione disulfide	-0.006	ns	**0.378**	**0.021**	-0.265	ns
5-Oxoproline	-0.231	ns	**-0.455**	**0.005**	0.028	ns
S-Glutathionyl-L-cysteine	-0.128	ns	**0.326**	**0.049**	-0.279	ns
Ascorbate	-0.088	ns	**-0.473**	**0.003**	0.274	ns
gamma-Glutamyl-Se-methylselenocysteine	-0.301	ns	**-0.422**	**0.009**	-0.028	ns
gamma-L-Glutamyl-D-alanine	-0.245	ns	**-0.432**	**0.008**	-0.013	ns
(5-L-Glutamyl)-L-glutamine	-0.015	ns	**-0.466**	**0.004**	0.283	ns
Dimethylglycine	-0.226	ns	**-0.449**	**0.005**	0.126	ns
Phosphoserine	-0.130	ns	**-0.396**	**0.015**	0.087	ns
Ornithine	**-0.328**	**0.048**	**-0.552**	**<0.001**	0.061	ns
L-Citrulline	**-0.341**	**0.039**	**-0.531**	**<0.001**	0.008	ns
N-Acetylneuraminate	-0.127	ns	**-0.378**	**0.021**	0.166	ns
alpha-D-Glucosamine 1-phosphate	-0.213	ns	**-0.350**	**0.033**	0.024	ns
Homocarnosine	**-0.456**	**0.005**	**-0.426**	**0.009**	-0.217	ns
Carnosine	-0.262	ns	**-0.512**	**0.001**	0.036	ns

Creatine	-0.177	ns	**-0.355**	**0.031**	-0.011	ns
Creatinine	-0.169	ns	**-0.410**	**0.012**	0.057	ns
trans-4-Hydroxy-L-proline	-0.241	ns	**-0.380**	**0.020**	0.018	ns
Quinolinic acid	**-0.332**	**0.044**	**-0.505**	**0.001**	0.021	ns
Anthranilate	-0.033	ns	**-0.352**	**0.033**	0.228	ns
L-Adrenaline	-0.235	ns	**-0.415**	**0.011**	0.060	ns
Ethanolamine phosphate	0.012	ns	**-0.329**	**0.047**	0.281	ns
N-Methylethanolamine phosphate	0.198	ns	**0.377**	**0.021**	0.001	ns
Acetylcholine	-0.084	ns	**-0.325**	**0.050**	0.231	ns
acyl-C20:4	0.303	ns	**0.416**	**0.011**	-0.052	ns
Butanoic/Butyric acid	-0.146	ns	**-0.395**	**0.015**	0.208	ns
Hexanoic acid (caproate)	**0.444**	**0.006**	0.227	ns	**0.349**	**0.034**
Dodecanoic acid (lauric acid)	**0.340**	**0.039**	0.095	ns	0.273	ns
Tetradecanoic acid (myristic acid)	**0.344**	**0.037**	0.300	ns	0.137	ns
Hexadecanoic acid (palmitic acid)	**0.411**	**0.011**	**0.357**	**0.030**	0.126	ns
Dodecanedioic acid	0.158	ns	**0.464**	**0.004**	-0.242	ns
DG(16:0;16:0)	-0.206	ns	0.043	ns	**-0.439**	**0.007**
DG(16:0;18:0)	-0.026	ns	**0.357**	**0.030**	-0.272	ns

DG(P-8:0;18:4)	0.201	ns	-0.155	ns	**0.376**	**0.022**
FA(14:0)	0.315	ns	**0.342**	**0.038**	0.039	ns
FA(19:0)	-0.142	ns	0.167	ns	**-0.337**	**0.042**
FA(20:0)	**0.358**	**0.029**	**0.325**	**0.050**	0.065	ns
FA(16:0)	**0.423**	**0.009**	0.271	ns	0.113	ns
FA(18:0)	**0.413**	**0.011**	0.287	ns	0.126	ns
Hex2Cer(d18:1;24:1)	-0.238	ns	0.144	ns	**-0.377**	**0.021**
LPC(20:4)	-0.013	ns	**0.337**	**0.041**	**-0.345**	**0.036**
LPC(P-24:1)	**-0.347**	**0.036**	-0.184	ns	-0.309	ns
LPC(18:3)	0.195	ns	**0.534**	**<0.001**	-0.178	ns
LPC(18:2)	0.114	ns	**0.347**	**0.035**	-0.197	ns
LPC(19:2)	0.144	ns	**0.401**	**0.014**	-0.202	ns
LPE(20:3)	-0.037	ns	**0.424**	**0.009**	**-0.371**	**0.024**
LPE(18:2)	0.029	ns	**0.363**	**0.027**	-0.280	ns
LPE(22:4)	0.026	ns	**0.351**	**0.033**	-0.221	ns
LPE(22:5)	0.022	ns	**0.413**	**0.011**	-0.268	ns
LPI(O-23:7)	-0.055	ns	**-0.341**	**0.039**	0.039	ns
LSM(d18:0)	0.219	ns	-0.264	ns	**0.432**	**0.008**

PC(36:4)	0.123	ns	**0.349**	**0.034**	-0.145	ns
PC(33:1)	0.095	ns	**0.326**	**0.049**	-0.187	ns
PC(O-38:3)	-0.293	ns	0.120	ns	**-0.394**	**0.016**
PE(36:1)	0.129	ns	**0.335**	**0.043**	-0.154	ns
PG(32:0)	**0.370**	**0.024**	0.316	ns	0.159	ns
PG(36:1)	0.101	ns	**0.334**	**0.044**	-0.181	ns
PG(16:0;18:1)	0.159	ns	**0.325**	**0.049**	-0.105	ns
TG(18:0;18:0;18:0)	-0.032	ns	**0.359**	**0.029**	-0.269	ns
TG(29:0;16:1)	**-0.427**	**0.008**	-0.054	ns	**-0.368**	**0.025**
TG(27:0;19:2)	0.232	ns	**0.363**	**0.027**	0.028	ns
TG(14:0;16:0;16:0)	0.197	ns	-0.281	ns	**0.373**	**0.023**
TG(15:0;16:0;16:0)	0.297	ns	-0.095	ns	**0.394**	**0.016**
TG(16:0;16:0;18:0)	**-0.360**	**0.029**	-0.106	ns	-0.294	ns
TG(24:0;16:0;16:0)	0.158	ns	-0.139	ns	**0.329**	**0.047**
TG(12:0;12:0;12:0)	-0.015	ns	-0.293	ns	**0.329**	**0.046**
TG(14:0;16:0;18:0)	0.271	ns	-0.261	ns	**0.461**	**0.004**

ns, not significant; FEV1, Forced Expiratory Volume in 1 s percentage predicted; FVC, Forced Vital Capacity percentage predicted; DG, diacylglycerol; FA, fatty acid; LPC, Lysophosphatidylcholine; LPE, Lysophosphatidylethanolamine; LPI, Lysophosphatidylinositol; LSM, Lysosphingomyelin; PC, Phosphatidylcholine; PE, Phosphatidylethanolamine; PG, phosphatidylglycerol; TG, triglyceride.
