# Supplementary material for: An integrated metabo-lipidomics profile of induced sputum for the identification of novel biomarkers in the differential diagnosis of asthma and COPD
Source: J Transl Med. 2024 Mar 23;22:301. doi: 10.1186/s12967-024-05100-2 (PMC10960495; doi:10.1186/s12967-024-05100-2)
Supplement: Supplementary file 2 — Additional file2: Figure S1. Heatmap illustrating the relative abundance levels of all the metabolites and lipids in asthmatic compared to COPD patients. Red indicates an increase in abundance, while blue a decrease. The columns and rows in the heatmap correspond to the experimental samples and the compounds, respectively. [file 12967_2024_5100_MOESM2_ESM.pdf]

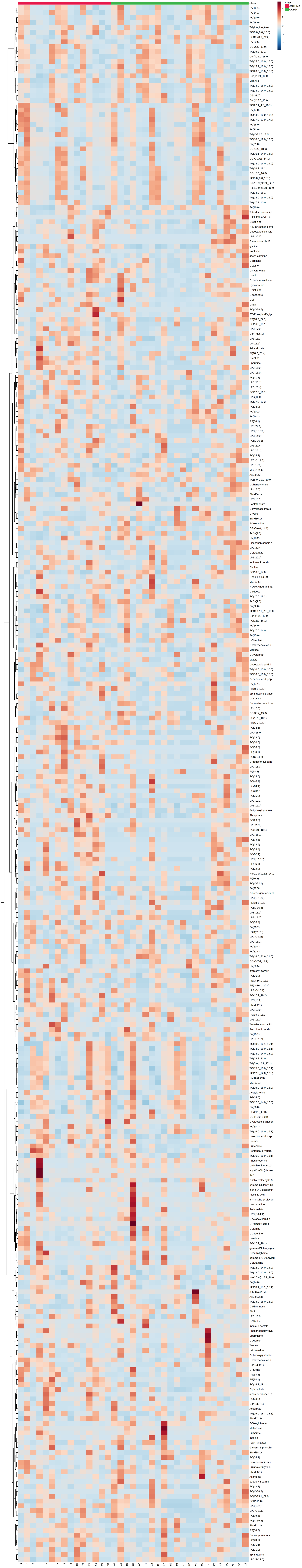
class
ASTHMA
COPD
